# A Boolean Seven-Layer Platelet Decision Model Models Consistently the Logic and Semiquantitatively the Dynamics of Platelet Activation and Inhibition

**DOI:** 10.34133/csbj.0228

**Published:** 2026-09-30

**Authors:** Özge Osmanoglu, Johannes Balkenhol, Juan Prada, Martin Kaltdorf, Maral Afshar, Sebastian Mammen, Katrin G. Heinze, Stepan Gambaryan, Harald Schulze, Thomas Dandekar

**Affiliations:** ^1^ Department of Bioinformatics, Biocenter, Julius-Maximilians-Universität Würzburg (JMU), 97074 Würzburg, Germany.; ^2^ Rudolf Virchow Center, Center for Integrative and Translational Bioimaging, Julius-Maximilians-Universität Würzburg (JMU), 97080 Würzburg, Germany.; ^3^ Department of Computer Science, Chair IX, Julius-Maximilians-Universität Würzburg (JMU), 97074 Würzburg, Germany.; ^4^ Sechenov Institute of Evolutionary Physiology and Biochemistry of the Russian Academy of Sciences, Saint Petersburg 194223, Russia.; ^5^ Institute of Experimental Biomedicine, Chair I, University Hospital Würzburg, 97080 Würzburg, Germany.; ^6^ EMBL, Molecular Systems Biology Unit, 69117 Heidelberg, Germany.

## Abstract

A platelet decision model integrates available experimental data to consistently model the Boolean logic in line with all available pathophysiological experimental platelet data. Due to insufficient kinetic data on involved protein networks, we semiquantitatively describe the dynamics of platelet activation and inhibition, i.e., the order of events and the relative strength of receptor activation or protein phosphorylation. The Boolean network model (76 nodes, 115 interactions) incorporates 7 layers of platelet signaling and regulatory feedback loops: (a) platelet activation stimuli resulting from vascular injury, (b) the platelet receptor layer (GPVI, GPIb-IX-V, PAR1/4, P2Y_1_/P2Y_12_, P2X1, TP, 5-HT2A, and IP) that receives signals, (c) central kinases (Src, Fyn, Lyn, PKC, PKA, and PKG) that converge diverse signals, (d) the translation of signaling into changes in platelet ion homeostasis, (e) alterations in platelet shape/adhesion, (f) the activation of other platelets, and (g) the formation of a platelet plug. Dynamic simulations based on curated omics, genetics, and functional data from experiments demonstrate network robustness by multiple and redundant positive feedback mechanisms. These allow rapid and irreversible aggregation and make the platelets resilient to even genetic knockouts (e.g., Trpm7). Signaling in chronic inflammatory conditions is modeled, such as in atherosclerosis, inflammatory arthritis, and aging, as well as inherited platelet disorders. We describe how acute decision responses can become maladaptive leading to chronic inflammatory decisions: This implies a risk of thrombo-inflammatory and cardiovascular diseases.

## Introduction

Best recognized by their roles in hemostasis, platelets are important players of immunity and inflammation. They recognize pathogens [1–4] and release inflammatory cytokines [5,6], recruit other immune cells to the infection site [7–12], and present antigens to adaptive immune cells such as B cells and T cells. However, their multifaceted physiological roles can also turn pathological, best exemplified in the context of immunothrombosis [13], in which activated platelets, mainly by pathogen recognition, lead to thrombotic complications and thrombo-inflammation. In this context, activation of platelets promotes inflammation and sustained inflammation leads to a pathological cycle of increased platelet activation [14,15].

In platelets, diverse processes and resulting signaling behavior are known:

Platelet pathological roles can manifest in various diseases such as cancer [16–19], in cardiovascular disorders such as atherosclerosis, myocardial infarction (MI), and cardiac arrest, and in sepsis, acute respiratory distress syndrome (ARDS), thrombosis, and autoimmune diseases [20–23].

In thrombo-inflammation, the lack of endothelial cell (EC) control on thrombosis and inflammation leads to hyperactivation of platelets [24,25]. ECs have an anti-thrombotic control over platelet function via various mechanisms. Likewise, ECs have an anti-inflammatory profile under healthy conditions. During thrombo-inflammation, inflammatory processes can shift the balance between pro- and anticoagulant properties of endothelium. Once the EC control is absent, platelets become hyperactive, and environmental stimuli can lead to their (self)-activating interaction with the endothelium and with various immune cells [24,25].

In atherosclerosis, which is a chronic inflammation disease, interaction of platelets with ECs and immune cells mediates thrombosis, inflammation, and angiogenesis [26,27]. Platelet adherence to the endothelium occurs through GPIbα and αIIbβ3 initiating the plaque formation [28]. Platelets further interact with the plaque via their GPVI receptors—earlier with laminin, and with collagen once the plaque ruptures. GPVI signaling is essential in atherothrombosis, and its inhibition was shown to inhibit thrombus formation [29]. Platelets do not contribute to the chronic inflammation in atherosclerosis only by their thrombotic function. They also recruit neutrophils and promote the adhesion of monocytes to the atherosclerotic endothelium and release cytokines and chemokines, increasing inflammation (such as CD154, IL1β, and CCL5 [24] and CXCL4, CXCL5, and MIF [28]). Platelet interaction with leukocytes and ECs is mediated mostly by platelet P-selectin [24], e.g., binding to PSGL1 on neutrophils, mediating interactions between platelet HMGB1 and neutrophil RAGE, and between platelet GPIbα and neutrophil integrin aMβ2 (MAC1) promoting neutrophil recruitment and NETosis. In return, neutrophils further activate platelets by the antimicrobial cathelicidin LL37/CRAMP they secrete after stimulatory platelet GPVI signaling [28].

What leads to atherothrombosis (plaque rupture) is continuous low-grade inflammation, during which ECs secrete von Willebrand factor (VWF) that promotes platelets’ adhesion by their GPIb receptor (GPIb-IX-V complex) activating platelet signaling. Platelet activation, in return, ends in release of factors like CD40 ligand, which induces ECs to produce more reactive oxygen species (ROS) and inflammatory molecules such as IL1β, so the ECs express more adhesion proteins promoting the adhesion of neutrophils and monocytes to the endothelium. At the same time, activated platelets also release PF4 and more ROS.

Nox1, but not Nox2, mediates GPVI-induced ROS production. Collagen-induced ROS production and aggregation are Nox1-dependent. Both Nox1 and Nox2 regulate collagen-induced thrombus formation at arterial shear [30]. Nitric oxide (NO) mediates inhibitory platelet signaling via guanosine 3′,5′-monophosphate (cGMP) [31]. When NO is scavenged by ROS, the absence of inhibitory signaling leads to more platelet activation and aggregation [32].

Another chronic inflammatory disease in which platelets show increased activity is inflammatory arthritis (IA). This activity is attributed to adenosine diphosphate (ADP) signaling. ADP normally activates platelets by binding to G protein-coupled receptors P2Y_12_ and P2Y_1_. Downstream signaling leads to platelet activation and release of factors including CD40 ligand (CD40L), which is also induced by increased tumor necrosis factor-α (TNF-α) levels in IA. CD40L, in return, induces ECs to produce and secrete more pro-inflammatory factors and leads to the activation of more platelets by inhibiting the ecto-ADPase of ECs; this results in higher amounts of available ADP in the bloodstream [33]. High levels of TNF-α are also observed in chronic inflammation during aging and is secreted by monocytes [34]. In return, activated platelets promote production of more cytokines by monocytes. Such platelets express surface proteins that increase the reactivity of even more platelets [35]. These platelets also showed higher surface expression of GPIbα and GPIX compared to platelets from young mice [34]. High levels of TNF-α asserts its effects by metabolic programming of the platelets, which leads to changes in pathways involved in mitochondrial function. These effects of TNF-α were shown to be on thrombopoiesis and megakaryopoiesis rather than platelet signaling directly [36].

Moreover, specific types or signatures of platelet activation have already been modeled in detail, e.g., Cognasse *et al*. [37] model platelet activation signatures in response to diverse immune and hemostatic agonists. Platelets from 10 healthy donors were stimulated under 7 conditions, including TRAP (PAR1), AYPGKF (PAR4), ADP, collagen, sCD40L, fibrinogen, and a control. A total of 47 markers—encompassing membrane proteins, soluble mediators, and intracellular signals—were analyzed. A reduced panel of 6 markers (AKT, CD40L, CD62P, PKC, RANTES, and TSLP) enabled identification of the stimulus with 86.8% accuracy. Van Holten *et al*. [38] modeled similar release profiles following specific PAR1 or PAR4 stimulation of platelets, showing that both may induce release of α-granules in a similar way. Kamykowski *et al*. [39] showed by quantitative immunofluorescence mapping low functional coclustering of proteins within platelet α-granules. Moreover, Zhou *et al*. [40] did intelligent classification of platelet aggregates by agonist type including high-throughput imaging flow cytometry of blood cells to differentiate different types of agonists.

In this study, we present a Boolean platelet decision model of the different decision layers of the platelet that integrates available experimental data to consistently model the logic of the complete platelet network and semiquantitatively model the dynamics of platelet activation and inhibition. Our model hence focuses on an integrated, complete model of all involved proteins in platelet reactions and the decision process in the network.

This is the strength of the model, predicting correctly the order of events and the relative strength of receptor activation or protein phosphorylation, while a detailed quantitative and kinetic model would require vastly more data on the kinetics of each protein.

Hence, it is also seen as complementary to detailed and fully quantitative models of specific activation processes [37,40], including the mechanisms of platelet transition between different phenotypes [41], or signatures to recognize an activation state [37,40] or specific quantitative modeling efforts [42,43] including specific platelet responses [44] or severe acute respiratory syndrome (SARS) infection-stimulated hyperactivity [45].

With our Boolean network models, we explore the mechanisms behind rapid, precise platelet decisions in balancing activation and quiescence, which is essential for hemostasis. We employ Boolean models to simulate platelet behavior under various physiological and disease conditions. The model was carefully validated regarding correctly predicting the right Boolean logic network state according to available datasets, functional studies, and platelet protein interactions. This resulted in a 7-layer decision model of platelet signaling. This now allows us to study in detail how signals travel in the platelet and identify key signaling events in physiological and pathological conditions. The model reveals how acute responses can become maladaptive in chronic inflammatory states, thereby increasing the risk of thrombo-inflammatory and cardiovascular diseases. We study the switch from hemostatic to thrombotic platelet functions, which can be described as a “decision-funnel”, i.e., this switch can be visualized from a broad set of network states in our Boolean network. This makes platelet response robust but also irreversible when committed beyond a signaling threshold. Moreover, the models allow us to explore novel and known pharmacological intervention strategies and how they are affecting the platelet network logic.

The comprehensive platelet decision network represents and integrates (a) the central activating cascade of the platelet [46–50], (b) the inhibitory cascade of adenosine 3′,5′-monophosphate (cAMP) [43] and cGMP [42], (c) modulation by calcium and magnesium signaling including TRPM7 [51], (d) models on hyperactivity of platelets in Aspergillus [52] or severe acute respiratory syndrome coronavirus 2 (SARS-CoV-2) infection [44], as well as (e) tumor-promoting activity in cancer [45], and (f) platelet hemostasis kinetics [41].

## Results

### Network model of platelet central cascade

The platelet central cascade for activation (CC) represents major players in platelet signaling and is modulated by cross-talk, such as modulatory proteins as well as drugs targeting these. In the comprehensive Boolean network, signaling processes were integrated as specific protein nodes and cascades and their relationship (activation, inhibition) depicted as directed interactions.

We assembled our model as follows:

We started with the CC, based on previous work on the central activating cascade of the platelet as systems biologically defined in terms of the key Boolean network controlling activation and modulatory protein interactions [47–50]. For details on how each protein node was incorporated and modeled, as well as the corresponding references, see Table S1. There are a total of 99 data references for all interactions and nodes modeled.

Hence, only protein nodes or output components were included if there was a reference for these available, and the starting references on the CC and its modulation provided a first backbone (see Table S1).

We then added more information to get a comprehensive Boolean model on the platelet decision process. Whenever the Boolean logic changes by new nodes or modifying connections, the overall network and its behavior changes. Using the connectivity of the Boolean network (also called “topology”) as input data, we can use dynamic software for interpolating between the ON and OFF states of the model and to get a more realistic model of the cellular responses, for instance, how the phosphorylation of Src changes after activation. For a detailed quantitative model of the platelet, the kinetic parameters would have to be collected, which would require a huge amount of data. Instead, the dynamic interpolation software Jimena applied uses heuristics to estimate the kinetics according to the Boolean logic of the network. These can be e-functions (software SQUAD; [53]) or e-functions plus further approximation functions (software Jimena; [54]). We used both approaches. The big advantage of this method is that the simulation covers the dynamics of the platelet responses according to the Boolean logic; the clear disadvantage is that such a model is only semiquantitative, i.e., only order relations apply. One knows only which protein kinase is more strongly or weakly activated, what comes first, and what comes later in the network. However, the simulation output including the dynamic shape depends only on the Boolean network topology. The parameterization follows only a heuristic (the e-functions, automatically input by the software) and changes only if the Boolean topology changes. Using the same topology and the same software, you get always the same responses and parameter settings that are important for the reproducibility of reported results.

One can then in a third step use data-driven modeling to be more accurate—in particular, if there are data available for the degree of activation. This is always indicated in the cited publications if done so.

Next, we considered for the Boolean network the central cascade up to first-, second-, and third-degree neighbors. This has been modeled in [46]. The key Boolean switches from this extension are also included in the model (see Table S1). Similarly, the inhibitory cascade around cAMP [43] is incorporated into our model as well as the later extension considering modulation and input by cGMP signaling [42] (Table S1). Finally, we also consider the modulation by calcium and magnesium signaling including Trpm7 for our Boolean network, as investigated by Gotru *et al*. [51] (see Table S1 for details).

To extend coverage of pathophysiological states, we included key modulatory interactions from our models on hyperactivity of platelets in the presence of Aspergillus spores [52] or SARS-CoV-2 infection [44] as well as network signals involved in tumor-promoting activity in cancer [45] and platelet hemostasis kinetics [41].

Taking all this together, we derived a detailed, 7-layer model of platelet signaling. Detailed references confirming the connectivity of the Boolean network are given in Table S1. The interactions, including activation inhibition, have been optimized according to recently available experimental studies and data.

The updated and comprehensive model of the central signaling cascade of the platelet incorporates different regulatory mechanisms of the platelet function (Fig. 1).

**Fig. 1.**
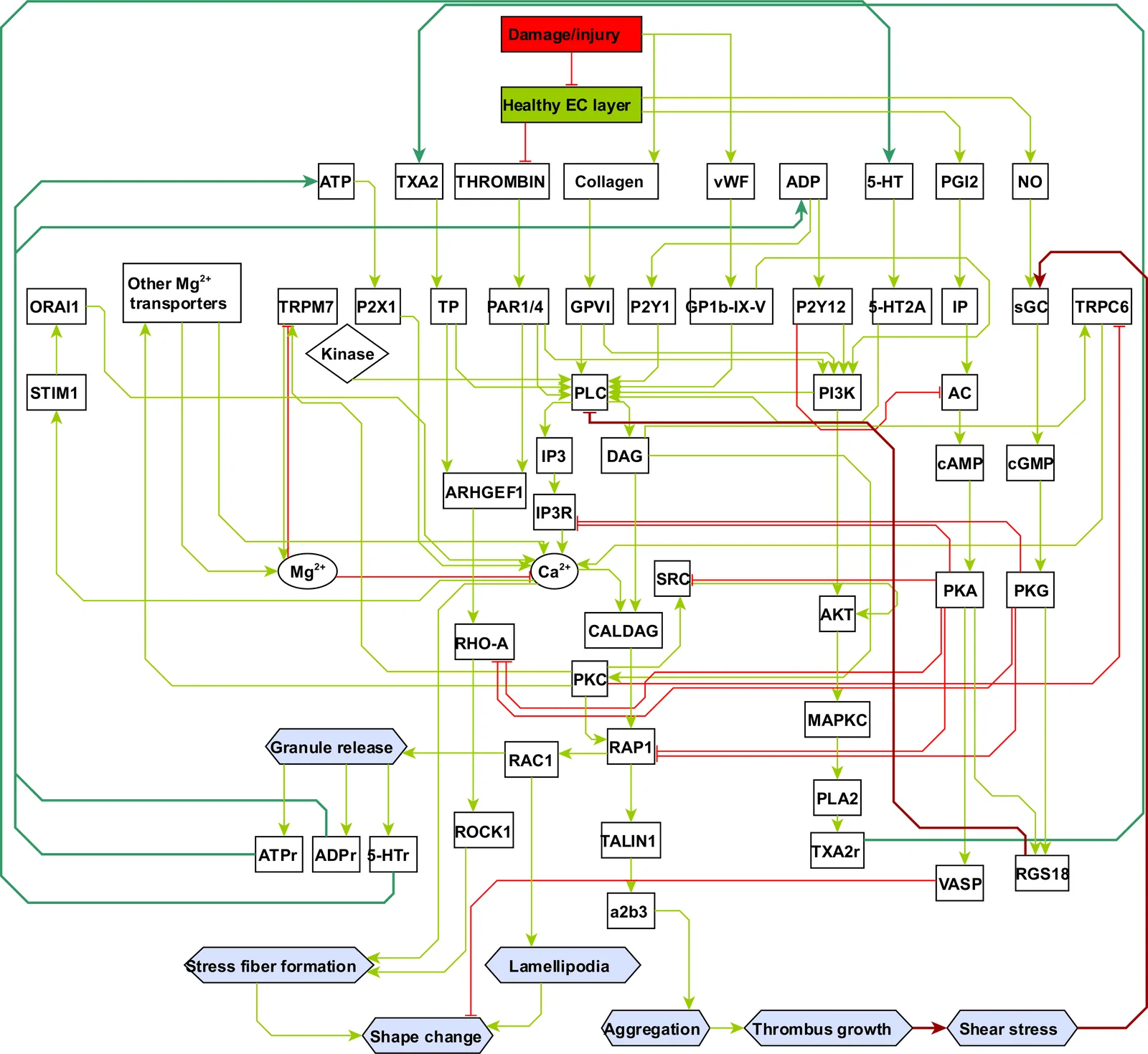
Cell signaling model of platelet activation. White boxes represent proteins. Their input from other proteins by interactions is shown as activating (green arrows) or inhibitory (red blunted arrows). The resulting cellular functions are shown as violet hexagons (e.g., shape change). Activating interactions in signal amplification are shown in thick green arrows (positive feedback), while internal and external brakes are shown in thick red arrows (negative feedback).

Calcium plays a crucial role in the platelet activation cascade, influencing key processes such as granule release, stress fiber formation, lamellipodia extension, and shape change [55]. Protein kinases PKA and PKG contribute to the regulation of platelet activation by targeting key molecules such as RAP1, RHOA, IP3R, and SRC while promoting the activation of VASP and RGS18 [42]. The process of shape change necessitates the formation of stress fibers and lamellipodia, coupled with the inactivation of VASP [56]. Additionally, active PKC facilitates signal transmission to SRC, TRPM7, and Mg^2+^ transporters; however, the inactivation of PKC is essential for TRPC6-mediated Ca^2+^ transport [51]. This signaling model highlights the complex antagonistic interactions between Ca^2+^ and Mg^2+^ in platelets. Furthermore, phospholipase C (PLC) acts as a pivotal convergence point for various signaling pathways activated downstream of platelet surface receptors.

### The platelet central cascade reflects the impact of multiple diseases on platelet function

We integrated 4 available datasets to better model key signaling pathways of platelet function including and around the CC. A heatmap shows the expression of key nodes under 4 different conditions (Fig. 2). A previous analysis [44] revealed changes in gene expression of the platelet CC nodes across cancer, non-ICU (intensive care unit), and ICU COVID-19 patients compared to healthy controls, highlighting the differences and the similarities between the effects of different disease types on platelet function (Fig. S2). Among the up-regulated genes, GPIbß, encoding for the subunit of the GPIb-IX-V complex, stood out as the only gene consistently up-regulated across all 3 conditions—cancer, non-ICU, and ICU—compared to healthy patients, suggesting a potential central role in disease/inflammation-associated platelet functions.

**Fig. 2.**
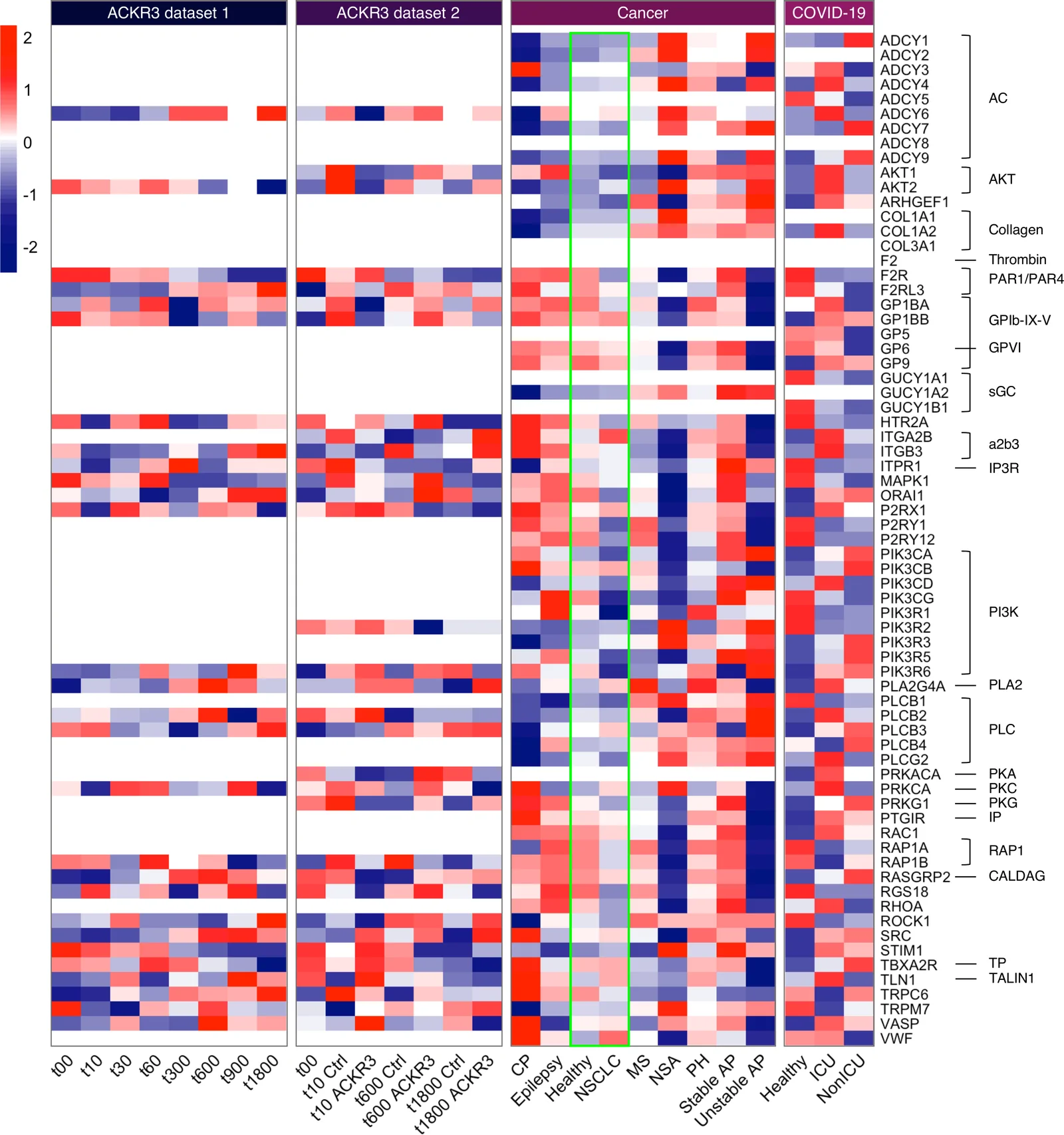
Omics data integration across 4 platelet datasets. Heatmap overview of gene expression and phosphorylation changes in platelet CC nodes across 4 datasets. The heatmap summarizes the gene expression and phosphorylation changes of platelet CC nodes in 4 datasets (all 4 datasets are given in Table S2): (1) ACKR3 dataset 1, a phosphor-proteomics dataset with 7 time points following ACKR3 stimulation; (2) ACKR3 dataset 2, a validation phosphor-proteomics dataset with 3 main time points and DMSO controls for each time point; (3) the Cancer dataset, an RNA-sequencing dataset from NSCLC patients; and (4) the COVID-19 dataset, an RNA-sequencing dataset from COVID-19 patients (Table S2). The analysis includes all relevant platelet CC node genes, including isoforms and subunits. Gene names are shown on the heatmap, with corresponding platelet CC node names provided on the right if they differ. The counts and intensities from each dataset were normalized and transformed into *z* scores separately, which makes direct comparisons between datasets inappropriate. Instead, this heatmap highlights within-dataset patterns of up-regulation and down-regulation.

In the context of non-small cell lung cancer (NSCLC) [45], several genes exhibited slight up-regulation [log_2_ fold change (log_2_FC) > 0.3], including ADCY2 [adenylate cyclase (AC) isoform], GUCY1A2 [soluble guanylate cyclase (sGC) isoform], TLN1 (Talin1), and VWF. Interestingly, ADCY2 and GUCY1A2 were absent from the COVID-19 datasets, while TLN1 and VWF showed no differential regulation in COVID-19 platelets. TLN1 is also of interest in the phosphor-proteomics datasets, where phosphorylation at S425 increases over time in dataset 1, and at S354, it shows a 3-fold increase in response to ACKR3 stimulation compared to the dimethyl sulfoxide (DMSO) control at 10 s (Fig. S2).

In contrast, several genes were consistently down-regulated in cancer and in COVID-19 patients: P2Y_1_, P2Y_12_, a regulatory subunit of PI3K (PIK3R1), and RGS18 were down-regulated in cancer, non-ICU, and ICU patients, respectively, indicating a shared pathway of platelet dysfunction or regulation in these disease states. ORAI1 was uniquely down-regulated in cancer, without significant changes in the COVID-19 datasets, suggesting a cancer-specific regulatory mechanism (Fig. S2).

Both PRKACA (PKA) and VASP were exclusively up-regulated in COVID-19 patients. PIK3R3 (regulatory subunit of PI3K) and TBXA2R (TP) showed increased expression across all 3 conditions, yet this up-regulation was only statistically significant in non-ICU patients, indicating that the regulation of these genes may vary in strength across disease severities. Similarly, while HTR2A and F2R (PAR1) were down-regulated exclusively in COVID-19 patients, they show mild down-regulation in cancer, suggesting that the down-regulation of these receptors may play a more pronounced role in the context of COVID-19. The analysis also highlights the regulation of GP5 (part of the GPIb-IX-V complex) and PIK3CG (catalytic subunit of PI3K) depending on the infection severity, where both were down-regulated in non-ICU patients, with PIK3CG showing a mild decrease in NSCLC platelets as well. ITGA2B (part of αIIbβ3) and P2RX1 are up-regulated in both cancer and ICU patients. Their expression is increased in non-ICU patients; however, this is only a trend, not statistically significant (Fig. S2).

Lastly, AKT1 is up-regulated exclusively in ICU patients, marking it as a potential marker of disease severity in the ICU cohort. There are no genes that are uniquely down-regulated in ICU patients. In the first phosphor-proteomics dataset, upon ACKR3 stimulation, we observed an increasing phosphorylation of TRPC6 (S836|S839), ROCK1 (T1101|S1105), F2RL3 (S366|S369), VASP (S322|S324), GPIBA (Y292|Y294), PLA2G4A (S727), and TLN1 (S425), especially toward later time points. A slight up-regulation was observed for SRC (S75), RASGRP2 (S394), and ADCY6 (S54). In contrast, phosphorylation of TBXA2R (S331), GPIBB (S191), F2R (S418), AKT2 (T451), STIM1 (S521), and P2RX1 (T386) decreased over time (Fig. S1A).

At the 10-s time point, compared to DMSO controls, phosphorylation of TLN1 (T354) increased 3-fold. In contrast, phosphorylation of GPIBA (T289), AKT1 (S129), AKT2 (T451), ADCY6 (S576), and GPIBB (S191|T193) was significantly lower. Additionally, phosphorylation of RASGRP2 (S576|S578) and TLN1 (S677) also decreased at this time point. At 600 s, phosphorylation of GPIBA (T289|Y292|Y294) increased 4-fold, while STIM1 (S512) showed a slight rise. At 1,800 s, phosphorylation of SRC (S17) and PLA2G4A (S437) was significantly higher compared to the DMSO samples (Fig. S1B).

### Platelet hemostatic function represented by dynamic simulations

The model reflects how platelets adapt their activation state to environmental conditions, addressing the complex challenges they continually face. This adaptability is demonstrated through dynamic simulations, which are based on connections and signal transduction in the platelet central cascade. The network represents the decision-making processes of platelets as a Boolean network, representing protein interactions as logical relationships of inhibition and activation among different proteins.

The quiescent (non-active) state of the platelet protein–protein interaction network serves as the foundation for all the subsequent dynamic simulations. This state, as depicted in Fig. 3A, provides a detailed visualization of both active and inactive pathways within the system. The network is organized into distinct layers.

**Fig. 3.**
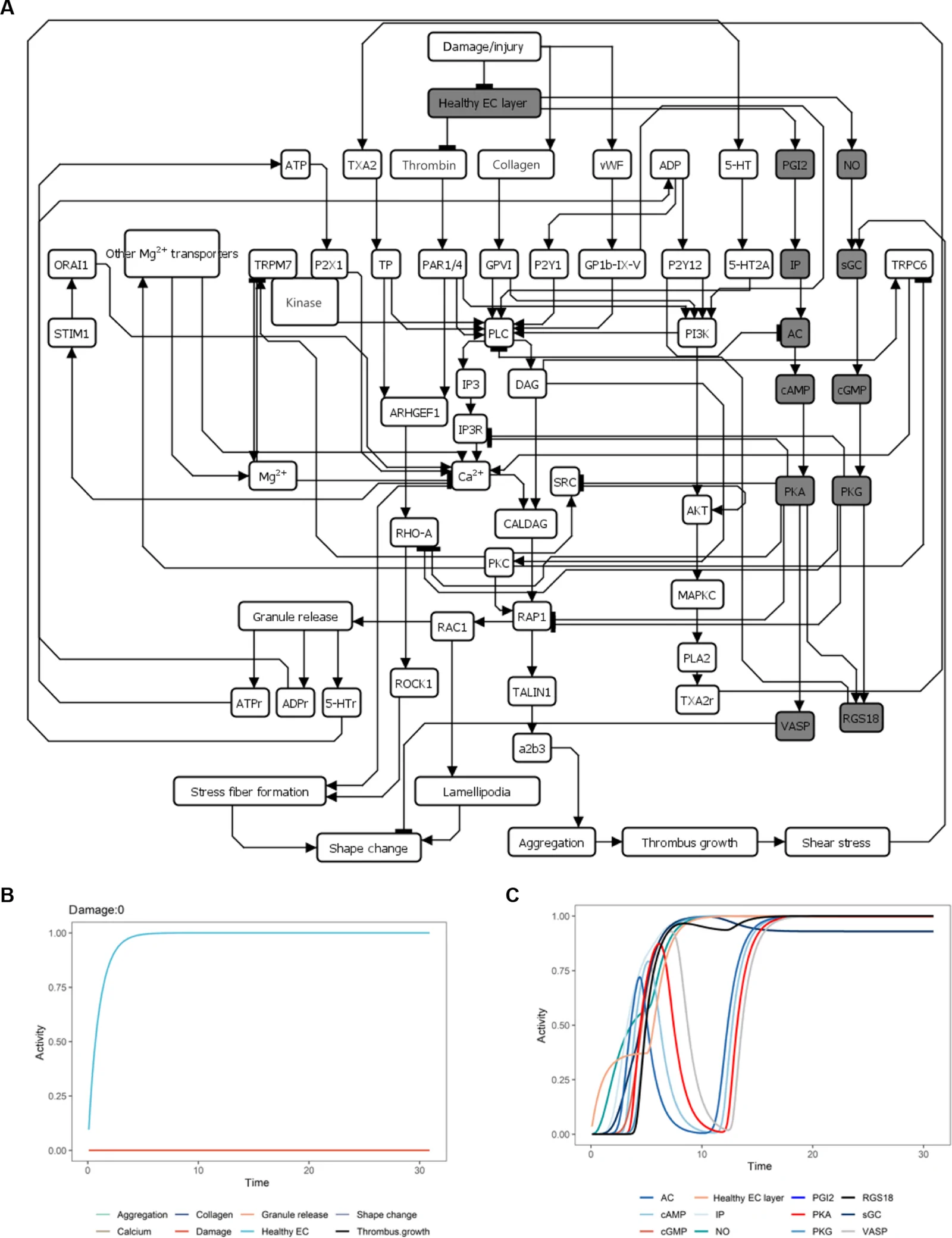
Quiescent state simulation. (A) Platelet signaling network at quiescent state. The activation of the nodes between values of 0 and 1 are shown: white: no activation; gray: high activation. Two input layers are indicated: Healthy EC layer keeping platelets quiescent in the absence of an injury and upstream damage/injury input. Signal processing proteins (internal layers) are indicated as rounded rectangles with uniprot identifiers (“nodes”), activation as arrows, and inhibition by blunted arrows. A feedback loop from behavior nodes to external input nodes is also depicted for amplifying platelet activation and self-inhibiting when the damage is repaired. The activation of the nodes changes over time. Thus, critical nodes can be monitored, indicating node and network behavior. (B) Platelet activation-related nodes in quiescent state maintained by the absence of damage. Blue, healthy endothelial cell (EC) state; orange, no damage signaling. (C) Platelet inhibition-related nodes in quiescent state are fully active [also shown in (A) as gray]. The healthy EC layer (orange tracking of activation intensity) releases PGI2 (dark blue tracing of amount of released PGI2). This activates cAMP (light blue tracing of activity) and cGMP (dark orange tracing of activity). This activates in turn PKA (red tracing) and PKG (blue tracing) dependent platelet inhibition signaling.

The first set of layers includes input nodes that represent environmental stimuli, such as damage to the EC layers, as well as platelet receptors that are designed to detect these changes in the environment. These input nodes are essential for gathering external information that influences platelet behavior [57,58].

The internal processing layers follow, where the external signals are integrated and interpreted, allowing the platelets to process complex information they receive from the environment. These layers are critical for determining the appropriate platelet response to varying conditions [59]. Finally, the output layers translate this processed information into observable behaviors, such as platelet shape change and aggregation. These behaviors are essential for the platelets to fulfill their role in clot formation and wound repair [31,59,60].

Additionally, the network includes a feedback loop from the behavior nodes back to the external input nodes, capturing the changes in the environment that are triggered by platelet activity itself. This feedback mechanism is crucial for allowing the platelets to continually adjust their behavior in response to ongoing environmental changes (Fig. 3A).

In the absence of injury, the endothelium serves as a non-adhesive layer that prevents platelet activation, maintaining platelets in a quiescent state. When platelet simulations are run without any external stimuli—such as damage to the endothelium—platelet signaling remains inactive (Fig. 3A). In this resting state, inhibitory pathways like cGMP, cAMP signaling, and RGS18 are fully engaged as self-regulatory mechanisms to prevent platelet activation (Fig. 3A).

This inhibition is primarily regulated by ECs, which secrete prostaglandin I2 (PGI2) and NO (Fig. 3B). Both molecules activate platelet inhibitory signaling through cyclic nucleotides, specifically cAMP and cGMP (Fig. 3C, also shown by Yau *et al*. [61]), ensuring that platelets remain inactive under normal conditions. Another mechanism by which ECs maintain platelet quiescence is through thrombomodulin, a protein that inactivates thrombin, thereby preventing clot formation ([62]; Fig. 3C, also shown by Yau *et al*. [61]). In our model, this process is represented by the inhibition of thrombin by the healthy EC layer [62].

In contrast to the quiescent state, platelet activation is rapidly initiated upon injury (Fig. 4). This transition is triggered by the exposure of subendothelial collagen and the interruption of inhibitory signaling from the healthy EC layer, persisting until the damage is repaired.

**Fig. 4.**
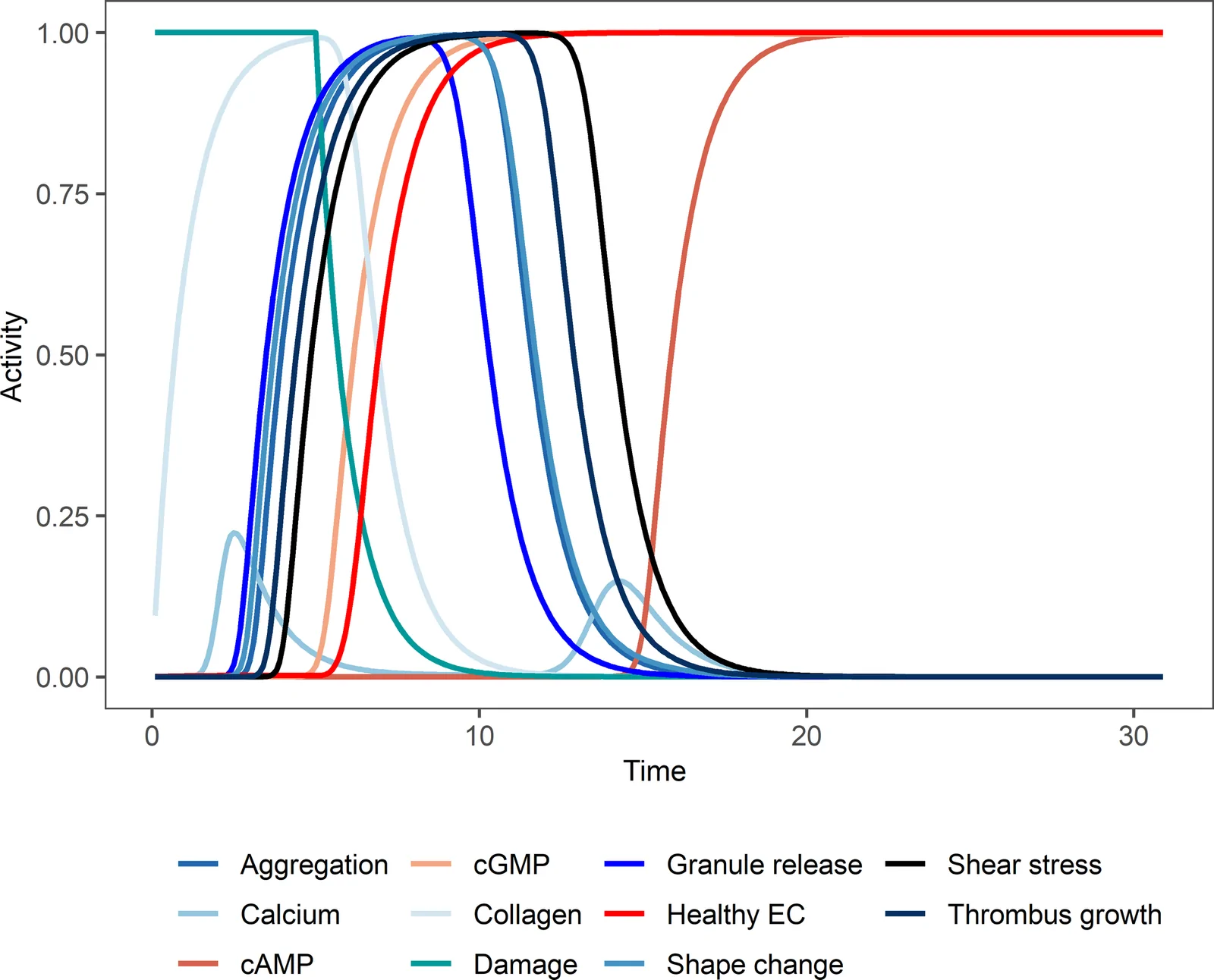
Damage to the EC layer immediately leads to collagen activation and platelet activation. The activation values over time of platelet activation markers (in shades of blue), platelet aggregation processes (black), and EC layer formation (red) are indicated.

### Dynamic simulations were validated extensively according to available data

For this, we combined a number of experimental and literature evidence (see also Table S1, 99 different data points and studies). For the different parts of the model, we highlight the following studies:

The core agonist–receptor interactions including collagen–GPVI, thrombin–PAR1/4, ADP–P2Y_1_/P2Y_12_, adenosine triphosphate (ATP)–P2X1, TXA2–TP, 5-HT–5-HT2A, VWF–GPIb-IX-V, and PGI2–IP are comprehensively reviewed by Yeung *et al*. [63].

Downstream convergence on PLC, with consequent generation of IP3 and DAG, activation of IP3R-mediated Ca^2+^ release, and store-operated Ca^2+^ entry via STIM1–ORAI1 and TRPC6, is supported by multiple independent genetic and pharmacological studies [51,63–66].

The role of TRPM7 kinase in modulating PLC phosphorylation and SOCE was directly demonstrated in Trpm7 knockout (KO) mice [51], while Mg^2+^ negative feedback on TRPM7 is well-characterized biochemically [67,68].

The inside-out integrin activation signaling spanning CalDAG-GEFI, RAP1, and talin-1 engagement of αIIbβ3 is supported by mouse genetic models, including talin-1 point mutations abolishing Rap1 binding [69,70].

The inhibitory cAMP/PKA and cGMP/PKG pathways, which suppress IP3R, RhoA, RAP1, and Src while activating VASP, are documented across multiple studies reviewed by Refs. [69,71,72]. A mechanosensory feedback loop in which shear stress generated by thrombus growth activates sGC–cGMP–PKG to limit further platelet activation was demonstrated by intravital imaging in cGMP-sensor mice [73].

At the omics level, the first comprehensive quantitative human platelet proteome identified nearly 4,000 unique proteins and solidly confirmed expression of the key signaling nodes including αIIbβ3, GPIb-IX-V, GPVI, and their signaling partners in this network [74].

Phosphor-proteomic profiling of resting human platelets identified 564 phosphorylation sites on over 270 proteins, including multiple novel PKA and PKG substrates [75]. Temporal quantitative phosphor-proteomics of ADP-stimulated platelets followed by iloprost-induced inhibition profiled over 4,700 phosphopeptides and directly mapped the reciprocal activation and inhibition dynamics of central network nodes including P2Y_1_/P2Y_12_ downstream targets, cytoskeletal regulators, and guanine nucleotide exchange factor (GEF)/GTPase-activating protein (GAP) proteins [76].

Collectively, these various lines of evidence support the structure and directionality of the network.

Note again that we have a Boolean decision network here as basis for all simulations; hence, there are key nodes involved in the decision process—i.e., receptors and central kinases such as Src—and the connectivity of the network identifies such hub nodes with many connections. However, we have insufficient detailed and large-scale data to provide a detailed, accurately parametrized, quantitative model of the platelet. Only then it would be possible to have a more dynamic analysis or comprehensive sensitivity or threshold analysis on the dynamics of the network responses.

Using dynamic simulations based on available datasets and studies mentioned above, we demonstrate that the robustness of the platelet signaling network was caused by multiple and redundant positive feedback mechanisms, which allow rapid and irreversible aggregation and make the platelets even resilient to genetic KOs (e.g., Trpm7). Simulating chronic inflammatory conditions like atherosclerosis, IA, and aging revealed aberrant platelet activation arising in all 3 conditions. In contrast, inherited platelet disorders such as GPVI deficiency, Glanzmann thrombasthenia (GT), and Bernard–Soulier syndrome have and show aberrant platelet activation from the start.

The initiation of platelet activation is driven by the binding of collagen to platelet receptors at the injury site [77]. Our model captures this appropriately, profiting from the iterative refinement steps according to available datasets mentioned above.

This initial adhesion is mediated by integrin α2β1 and glycoprotein GPVI (ITAM signaling), with the GPIb-IX-V complex also contributing to stable adhesion under high shear conditions by binding to VWF [63,78] (only GPVI is represented in Fig. 4). These interactions trigger downstream signaling via PLCγ (PLC) and PI3Kβ (PI3K), leading to platelet activation [63,78].

Activation of PLC results in increased cytosolic Ca^2+^, which activates Rap1B and Rac1, leading to cytoskeletal rearrangements and subsequent shape change. Platelets transition from their natural discoid shape to an extended form through the formation of lamellipodia and filopodia. Concurrently, Rac1 activation also promotes α-granule secretion, releasing agents like ADP, ATP, and serotonin [5-hydroxytryptamine (5-HT)] [79].

Granule release and TxA2 production amplify the platelet activation signal by engaging various G protein-coupled receptors (PAR1/4, P2Y_1_, P2Y_12_, P2X1, TP, 5-HT2A) and their associated G proteins. These signaling pathways lead to further activation of PLCβ, PI3Kγ, and ARHGEF1 and inhibition of AC, lifting the inhibitory effects of cAMP signaling [63,72,80–82]. This sequence of events activates αIIbβ3 (depicted as a2bb3 in the network), allowing it to bind extracellular ligands like fibrinogen or VWF, facilitating platelet aggregation.

The positive feedback mechanism via granule release ensures rapid platelet aggregation, crucial for responding swiftly to vascular injury. This robust mechanism, demonstrated to be resilient against Trpm7 KO (shown in Fig. 5), amplifies the initial activation signal, rapidly recruiting additional platelets [80] (shown in Fig. 6 for constant damage; in Fig. 7, for chronic inflammation; and in Fig. 8, for inherited disorders).

**Fig. 5.**
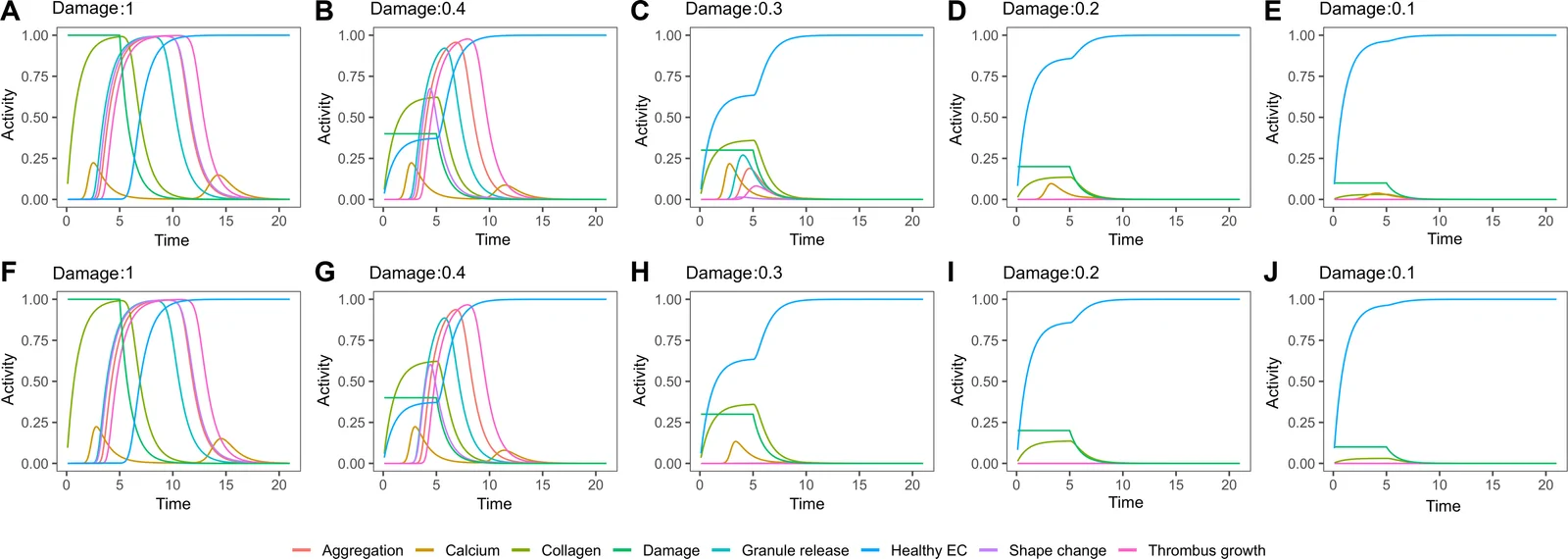
Trpm7 KO resilience of platelet signaling. (A) Trpm7^−/−^ simulation with external control layers. The Trpm7 kinase domain is set as constitutively active in WT [up: (A) to (E)] and 0 in Trpm7 KO [down: (F) to (J)]. Different damage input values stimulate platelet signaling in both conditions. Threshold analysis determined the intermediate value of collagen to be 0.45 for WT and 0.45 for Trpm7^−/−^ [defined as the minimum collagen stimulus required to reach a full irreversible aggregation (value 1)] and the lower value to be 0.35 for WT and 0.4 for Trpm7^−/−^ (the minimum collagen stimulus to activate the irreversible aggregation to a higher value than the collagen input).

**Fig. 6.**
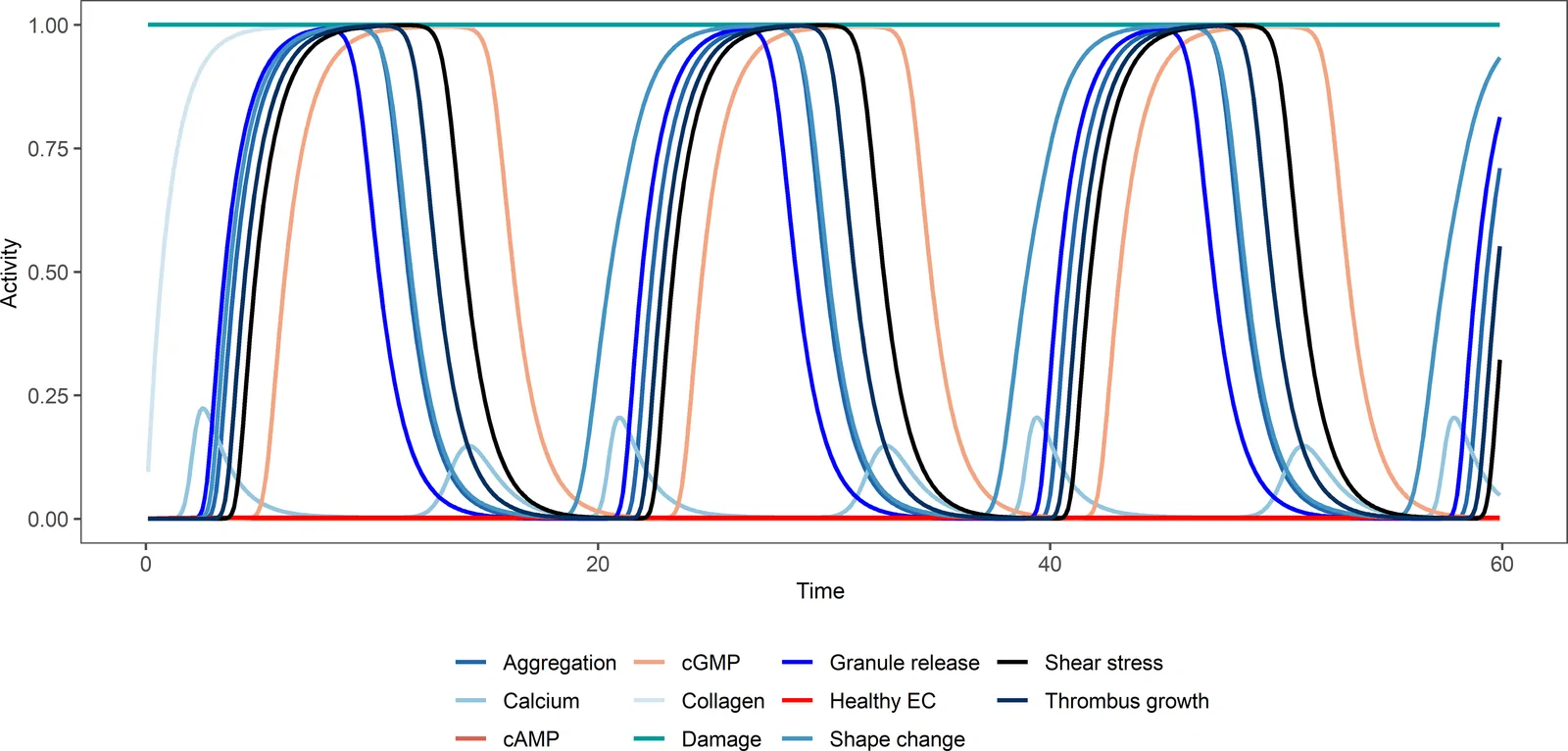
Constant damage (A) and EC absence (B) simulations. (A) Platelet activation persists, although some of the intrinsic self-inhibition mechanisms (e.g., RGS18-cGMP signaling) respond to platelet activation by trying to suppress it. However, continuous external input leads to repeated platelet activation (damage/injury: value 1 from 0 to 1,000). (B) Platelet activation persists similarly to constant damage simulation, although some of the intrinsic self-inhibition mechanisms (e.g., RGS18-cGMP signaling) respond to platelet activation by trying to suppress it. However, the lack of EC control leads to repeated platelet activation, even in the absence of damage (healthy EC: value 0 from 0 to 1,000).

**Fig. 7.**
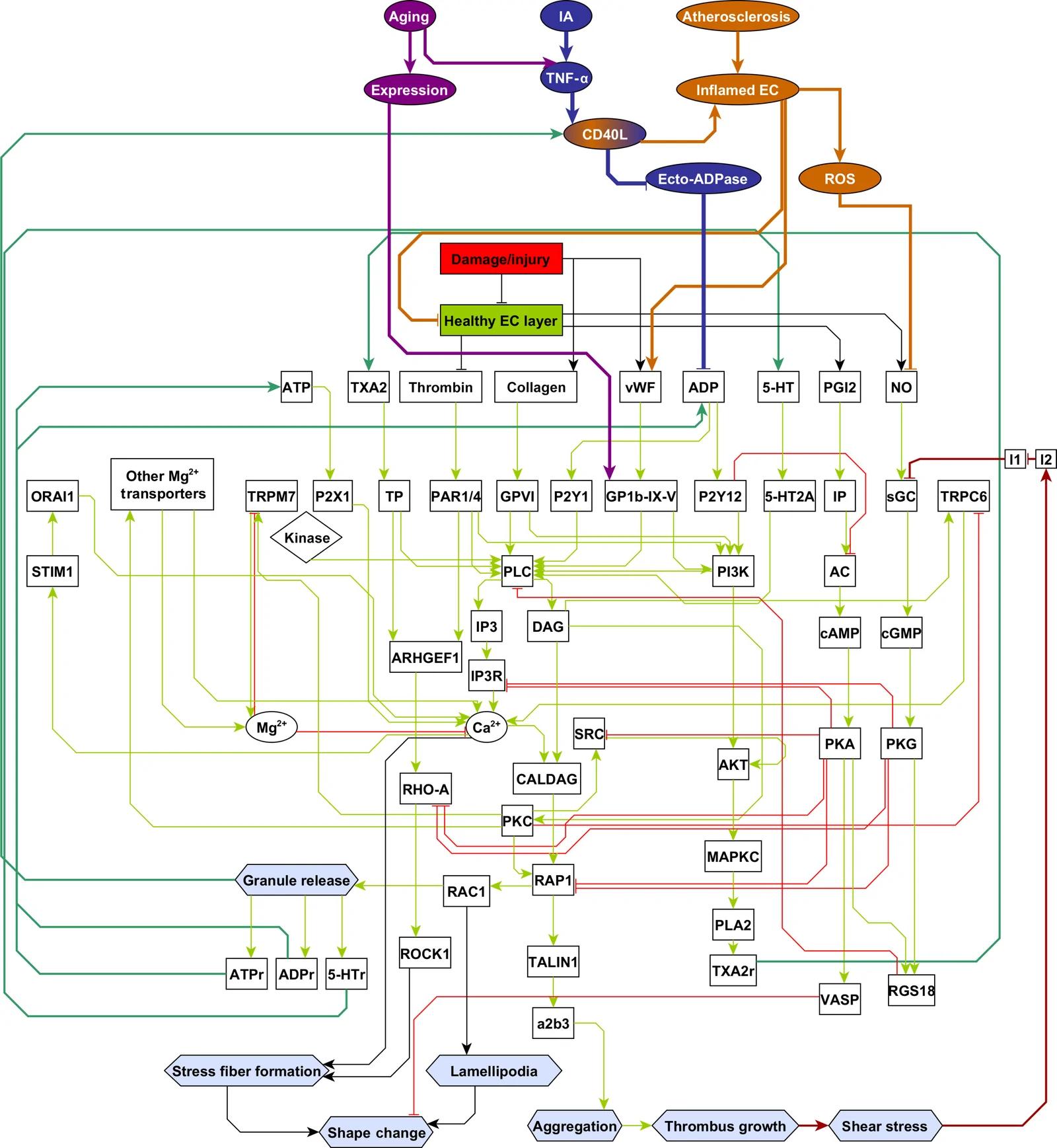
Chronic inflammation causes aberrant platelet activation. Overview of disease mechanisms integrated in the model. Effect of inflammation on platelet signaling is shown with a focus on atherosclerosis, inflammatory arthritis (IA), and aging. In atherosclerosis, ECs secrete VWF and promote platelet adhesion. In return, activated platelets release CD40L, which induces ECs to take on a more pro-inflammatory and pro-thrombotic profile, leading to more platelet activation, e.g., via inhibition of platelet-inhibitory cGMP signaling. In IA, ECs also show a pro-inflammatory and pro-thrombotic profile, and this increases platelet activation mainly via ADP signaling. Factors like CD40L inhibit the ecto-ADPase of ECs, promoting the ADP signaling even more. Lastly, in aging, although metabolic reprogramming is a main way of regulating platelets, we here only show the effects on signaling. This is exerted mainly via TNF-α secretion by monocytes and via increased expression of platelet adhesion receptors, like GPIb-IX-V.

**Fig. 8.**
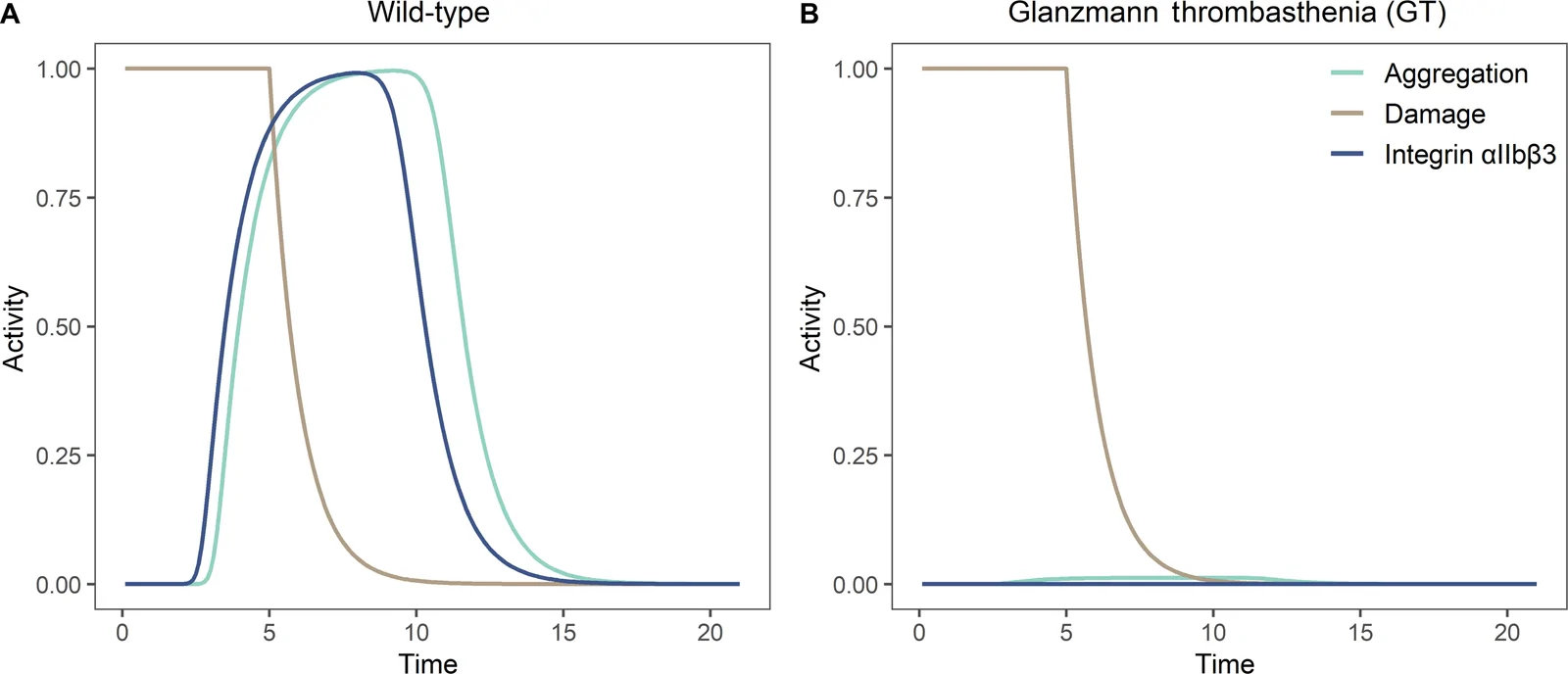
Inherited platelet disorders—loss-of-function simulations. (A to D) Simulation of platelet aggregation in response to collagen in (A) WT platelets, (B) platelets with GPVI deficiency, and response to damage in (C) WT platelets and (D) GT.

However, platelet aggregation is tightly regulated and does not continue indefinitely. Activation is terminated once the damage is repaired. This is achieved through internal and external feedback mechanisms. Internally, RGS18 acts as a self-regulating brake by inhibiting G protein signaling, a process typically activated by platelet inhibitors like cyclic nucleotides [83]. In our model, this internal mechanism is represented by the inhibition acting directly on PLC downstream of G protein signaling.

Externally, the mechanosensitive cGMP signaling pathway functions as an additional brake, inhibiting thrombus growth in response to increasing shear stress within the bloodstream as the clot size increases. This process involves the mechanical activation of guanylyl cyclase, which helps prevent excessive thrombus formation [84].

### Robustness of platelet decision-making relies on signal amplification through positive feedback loops

The robustness of the platelet signaling network is highlighted by the presence of positive feedback loops that ensure rapid and effective platelet aggregation. This resilience is evident in our model, which remains resistant to the effects of various KOs due to redundancy in platelet signaling. As an example, we studied Trpm KO (Fig. 7) [51].

Even when Trpm7 is knocked out, the platelet activation process remains largely unaffected, demonstrating that platelet reactivity is primarily driven by the extent of vascular damage (ranging from 1 to 0.1) rather than by the absence of Trpm7, as also shown by threshold analysis without external control (Fig. S3). Furthermore, the importance of external control layers in stabilizing the decision-making processes of the platelet network becomes particularly apparent when examining responses to damage in the absence of such external modulation. In scenarios where signal amplification or the nuanced context provided by the biological microenvironment is missing (Fig. S1), the impact of KO events becomes significantly more pronounced (Fig. S4). In these situations, platelet reactivity is still observed at certain levels of damage (e.g., between 0.4 and 0.2; Fig. 5A to E). However, these networks demonstrate increased sensitivity to Trpm7 KO, which leads to a diminished response to damage when external stabilizing influences are absent (Fig. 5F to J; Trpm7 KO threshold without external control: Fig. S7).

Consistent with the literature, the model predicts that TRPM7 loss does not abolish platelet aggregation but only slightly affects activation behavior. The threshold values were obtained through systematic threshold analysis. Specifically, we varied the collagen (damage) input across a continuous range and ran dynamic simulations for each value. The thresholds were then defined as follows: (a) the minimum input required to reach full, irreversible aggregation, and (b) the minimum input at which aggregation exceeds the initial stimulus. Using this approach, we observed only minor differences between wild-type (WT) and Trpm7 KO conditions, indicating that redundant pathways and feedback loops compensate for the loss of Trpm7, thereby preserving platelet activation.

The reader is reminded that we have a model of the Boolean network and the decision processes involved where we deduce the dynamics by simplified interpolation between ON and OFF states according to the network topology involved. We do not have an accurate parametrized quantitative model of the platelet, as this would require much more data on the kinetics. We hence mean here by “irreversible aggregation” only the committed decision to trigger platelet aggregation, not accurate measurement of the actual platelet aggregation.

All threshold values we describe are only emergent tipping points of the network dynamics identified by systematic simulation—they are not direct biological measurements or calibrated parameters.

### A shift in the decision funnel causes platelet hyperactivation

In thrombo-inflammation, the loss of EC control over thrombosis and inflammation results in heightened platelet activity.

ECs normally exert anti-thrombotic and anti-inflammatory influences. However, during thrombo-inflammation, the balance between pro- and anticoagulant properties of the endothelium can be disrupted, leading to sustained platelet activation and immune cell recruitment. In the absence of EC control, platelets become hyperactive and continuously interact with the endothelium and immune cells [24,25].

Our carefully set-up and refined simulations of the platelet decision network demonstrated this, too, showing a cyclic pattern of platelet activation in the persistent presence of procoagulant stimuli or when EC control is compromised (Fig. S6 provides an overview of disease mechanisms; Fig. 9). Next, we analyze different mechanisms of how this shift in balance happens and how the resulting chronic inflammation sustains platelet activation and increases the risk of thrombotic events and inflammation. We show this shift correctly having integrated the mechanisms behind inflammation caused by atherosclerosis, IA, and in aging (Fig. 7) and can *in silico* recapitulate the effects of chronic inflammation stimulating platelet aggregation (Figs. S9 to S11).

**Fig. 9.**
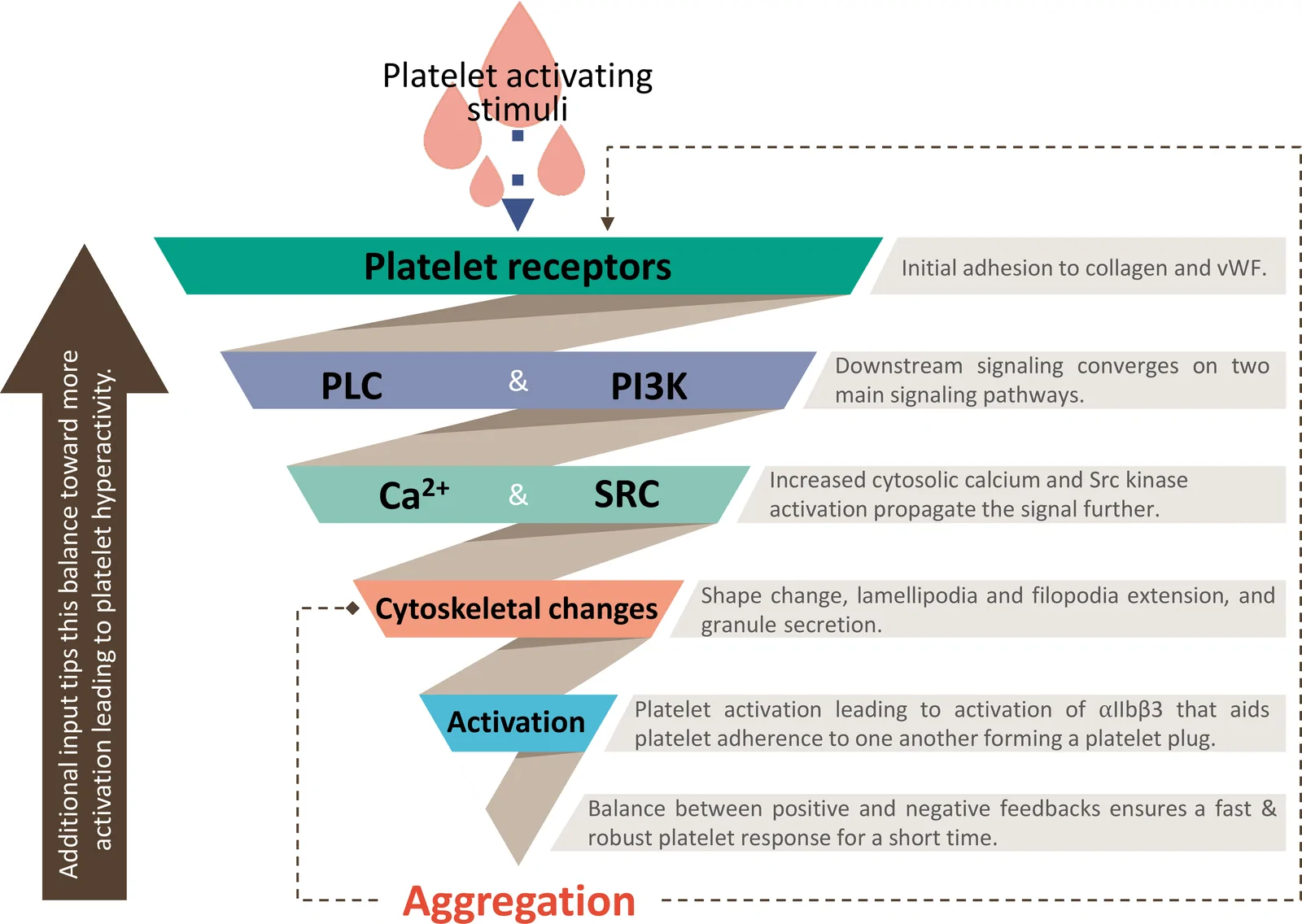
Decision funnel aggregated in several processing layers. Sensors (platelet receptors) measure the environmental change and give environmental feedback. In interconnected downstream cascades, the information is integrated step by step and translated into actions of effectors that induce cytoskeleton changes underlying the process of aggregation.

We modeled the effect of ECs during the low-grade inflammation in atherosclerosis by adding the mechanisms summarized above (Fig. 7).

Our carefully optimized model regarding the platelet decision nodes (for validation steps and data, see above) allows us to simulate different levels of atherosclerosis both in the absence of damage or injury and when there is a damage stimulus.

In all simulations, we started with a fully active “healthy EC layer” to simulate the changing balance between the healthy and inflamed EC layer. We introduced a damage stimulus at time point 10 to see the effects on a situation, where both inflammation and EC control are present at different levels.

In the absence of damage, our simulations showed atherothrombosis only at high levels of inflammation caused by atherosclerosis (atherosclerosis ≥ 0.7). Medium levels of inflammation (0.52 ≤ atherosclerosis < 0.7) led to aberrant platelet aggregation only in the presence of a damage stimulus. In other words, in the medium range of atherosclerosis, a damage stimulus was enough to shift the balance from healthy EC to inflamed EC. However, low-grade inflammation (atherosclerosis < 0.52) did not result in atherothrombosis even in the presence of a damage stimulus, which failed to disrupt a healthy EC layer and its anti-thrombotic profile (Fig. 7). We considered next the interplay between the IA-induced pro-thrombotic EC profile via ADP signaling and the platelet hyperactivation in our validated model (for datasets and validation, see above) and simulated the effects of different levels of IA on platelet aggregation (Fig. S11). Aging simulation results are shown in Fig. S12. We observed a similar trend as in atherosclerosis; however, IA exerted a stronger effect on platelet aggregation even at lower/medium levels (IA ≥ 0.58) and ended up in full activation of platelet aggregation even in the absence of a damage input (Fig. S9).

The reported values (e.g., 0.52, 0.58, and 0.70) were obtained through a systematic analysis of the semiquantitative Boolean model, not through calibration or optimization. Disease inputs were defined as continuous normalized variables (0 to 1), and we performed a systematic analysis by gradually increasing these values and running dynamic simulations of the network. For each input level, we monitored platelet aggregation and classified the system behavior (no aggregation, damage-dependent aggregation, or spontaneous aggregation). The thresholds correspond to the minimum input values at which qualitative transitions in behavior occur. Thus, they represent emergent tipping points of the network dynamics, determined operationally from simulations, and are model-specific and unitless, rather than directly fitted biological parameters.

Disease thresholds correspond to behavioral transitions: Example (atherosclerosis): <0.52 → no aberrant aggregation; 0.52 to 0.7 → aggregation only with damage; ≥0.7 → aggregation even without damage.

In our model, having refined the activation and interplay between the receptors according to the available datasets and knowledge on interactions, we can also show how aging shifts the signaling of platelet receptors GPIbα and GPIX, as well as inflammation through TNF-α levels.

Although metabolic reprogramming was not considered in our model, our simulations closely mirrored the results seen in platelet hyperactivation in IA-induced inflammation (Fig. S11).

### Inherited platelet disorders

Inherited platelet disorders easily disturb the platelet balance, majorly concerning the size of the platelets and their function [85,86]. The first type of platelet disorder can lead to low counts of platelets (thrombocytopenia) or high counts of platelets (thrombocytosis) and is mostly caused by mutations in genes associated with megakaryopoiesis and thrombopoiesis. On the other hand, diseases associated with hypofunctional platelets can happen due to mutations in major platelet receptors. The validated 7-layer platelet model well simulates the effect of such mutations for various genetic diseases. GPVI deficiency is associated with absent or severely reduced platelet aggregation in response to collagen and other GPVI agonists. In agreement with these observations, our simulations with inactive GPVI (Fig. S13) show the absence of aggregation in response to collagen (Fig. 8A and B).

Another well-known disease associated with mutations in platelet receptors is GT, in which mutations in αIIbβ3 genes lead to absent or severely reduced light transmission aggregometry (LTA) with all agonists (ADP, TxA2, collagen, thrombin) [87]. In agreement with these observations, our model correctly simulates the effect of αIIbβ3 loss of function, including the absence of platelet aggregation (Fig. 8C and D; full GT simulation: Fig. S15). Bernard–Soulier syndrome is caused by mutations related to genes coding for the subunits of GPIb-IX-V, and it is represented by the absence of ristocetin-induced platelet aggregation and reduced response to other agonists like collagen and ADP [88]. The effect of GPIb-IX-V deficiency was also reproduced by our simulations, in which platelet aggregation in response to VWF was completely lost, while we did not observe differences in collagen or ADP response (Fig. S8A to F; full Bernard–Soulier syndrome simulation: Fig. S14).

## Discussion

### Basis: A 7-layer model of the platelet decision network and its signaling agrees with available experimental data

Our simulation integrates previous, experimentally well-validated models on platelet signaling, but now integrates all this information into a Boolean network. It models platelet decision wiring in a detailed, unprecedented completeness regarding the network. As a result, we have a 7-layered systems-biological framework model with positive (activating) and negative (balancing) feedback loops. In particular, the following major datasets were specifically considered to test and validate our model.

The comprehensive platelet decision network represents and integrates now appropriately

(i) the central activating cascade of the platelet [46–50],
(ii) the inhibitory cascade of cAMP [43] and cGMP [42],
(iii) modulation by calcium and magnesium signaling including Trpm7 [51],
(iv) models on hyperactivity of platelets in Aspergillus [52] or SARS-CoV-2 infection [44], as well as
(v) tumor-promoting activity in cancer [45], and
(vi) platelet hemostasis kinetics [41].

The interactions, including activation and inhibition, have been optimized according to recently available experimental studies and data (see Table S1; a total of 99 data references for all modeled interactions).

At its core, it has the central intracellular platelet activation network. This includes the canonical pathways such as calcium-driven activation (Ca^2+^–CalDAG–RAP1), PLC signaling (receptor activation leading to PLC, which generates IP3 and DAG), PKC and Src signaling, the PI3K/Akt pathway, and the integrin activation cascade (Rap1–Talin– αIIbbβ3 leading to aggregation). It also retains the inhibitory cAMP/PKA axis, where PKA down-regulates key signaling components such as SRC, Rap1, and IP3R. Our model places the central activating cascade into a broader and more physiologically detailed framework.

First, it expands the range of receptors and activation pathways, incorporating additional stimuli such as thrombin (via PAR1/4), serotonin (5-HT receptors), and VWF interactions (GPIb-IX-V). This makes the model more comprehensive in terms of how platelets are activated under different biological conditions.

Second, it introduces an explicit extracellular and vascular context. It models factors such as endothelial regulation (e.g., NO and prostacyclin), vascular injury, collagen exposure, and shear stress from blood flow. This shifts the perspective from an isolated intracellular network to a context-dependent system where platelet behavior is influenced by its environment.

Third, the model incorporates feedback mechanisms driven by granule release. Activated platelets release molecules like ADP, ATP, and serotonin, which then feed back into the signaling network and amplify activation. This considers important dynamic, self-reinforcing loops in the platelet network logic.

Another important addition is a detailed representation of cytoskeletal regulation and shape change. Our model explicitly models pathways involving Rac1, Rho-A, and ROCK1, leading to structures such as lamellipodia and stress fibers. This links biochemical signaling to physical changes in platelet morphology. It also expands ion regulation beyond calcium, incorporating magnesium dynamics and additional ion channels such as TRPM7 and TRPC6. Furthermore, the model adds new signaling pathways, including the cGMP/PKG axis (mediated by NO) and mitogen-activated protein kinase (MAPK) signaling. These pathways provide parallel regulatory mechanisms and additional cross-talk with the core signaling network.

Finally, we extend the model to higher biological scales. Instead of stopping at platelet aggregation, it models downstream processes such as thrombus growth and its interaction with shear stress. This transforms the model from a purely intracellular signaling map into a multi-scale representation of platelet function in the context of interacting cells (e.g., other platelets and endothelium).

Taking all these data and signaling interactions together, we derive a detailed, 7-layer model of platelet signaling giving responses in accordance with all these datasets and well-validated signaling responses. The model offers the option to study in detail how signals are processed in the platelet over the 7 decision layers, including all proteins and their signaling status and changes. It further enables exploration and qualitative testing of pharmacological intervention strategies and assessment of the effect of other network changes (e.g., gene KO, genetic change, and pathophysiological conditions).

### Limitations: Only qualitative platelet decision-making modeled in hemostasis and thrombo-inflammation

Platelets are highly efficient at making rapid and accurate decisions, a capability that stems from the delicate balance between platelet activation and quiescence. This balance is crucial for maintaining hemostasis, and any disruption can lead to a shift toward a thrombotic state. To function properly, platelets must constantly monitor and adapt to changing conditions; failure to do so can have pathological consequences.

We modeled this platelet decision process using a 7-layer signaling network (Fig. 9). We focused on the network logic (Boolean network) and mirrored the decision processes in the network as close as possible to observation. However, this is only a semiquantitative model in which order relations hold (stronger, weaker activation; earlier, later event). Moreover, the Boolean/semiquantitative framework represents platelet signaling using normalized node activities between 0 and 1. While this approach is computationally efficient for systems-level analysis, platelet activation may become dose-, time-, shear-, and context-dependent if conditions get extreme for any of these parameters (e.g., high shear; dose-dependent drug behavior or combination of drugs). Hence, platelet behavior for such conditions is only qualitatively captured by our model.

The use of simplified disease input nodes is a conscious choice to keep the model both interpretable and aligned with our main objective.

The integration of cancer and COVID-19 datasets allowed us to identify which components of the platelet signaling network are consistently altered under these pathological conditions, and we incorporated these insights qualitatively by mapping and highlighting the affected nodes within the network.

For other conditions such as atherosclerosis, IA, and aging, where platelet-specific omics data were not available at the time of this study, we represented disease effects in a simplified manner. These were implemented as aggregated external input nodes, capturing the dominant upstream influences described in the literature, such as endothelial dysfunction, increased procoagulant signals (e.g., VWF and ADP), and inflammatory mediators.

Importantly, we modeled these effects at the top-most input layer of the system, rather than introducing disease-specific perturbations within the intermediate signaling cascade. This reflects our primary goal: to study the switch between platelet quiescence and activation, and to understand how different types and strengths of external stimuli drive this transition. By keeping the internal signaling network unchanged and modulating only the input layer, we can systematically assess how disease environments shift platelet decision-making toward sustained activation.

As a result, the model captures the key external, disease-driven mechanisms, how they influence platelet activation decision nodes, and their effect on aggregation dynamics. At the same time, it does not resolve detailed intracellular disease-specific mechanisms, gene-level regulation, or complex processes including quantitative comparisons such as metabolic reprogramming.

The Boolean model itself is accurate including involved nodes and their activation/inhibition state. A comprehensive parameter sensitivity analysis would require a fully quantitative model, which is beyond the scope and data available for this study.

In summary, the disease nodes in our network model of the decision process in the platelet serve as simplified, high-level representations of pathological conditions, enabling us to integrate available data where possible and, where data are lacking, to still capture the dominant external drivers of platelet activation in a consistent and interpretable framework.

The model is intentionally constructed as a reduced, systems-level representation of platelet signaling. As such, it prioritizes the main signaling pathways and feedback structures that determine activation outcomes, rather than incorporating all layers of biological complexity. Hence:

• Platelet heterogeneity is not modeled, as we treat platelets as a uniform population to focus on average signaling behavior.
• Secretion dynamics (α-granules versus dense granules) are simplified into aggregate granule release and feedback signaling, without distinguishing granule subtypes.
• Metabolic reprogramming (e.g., mitochondrial changes in aging) is acknowledged but not explicitly modeled, as our focus is on signaling rather than metabolism.
• Platelet–leukocyte interactions are captured only indirectly through inflammatory inputs, not as explicit cell–cell interaction modules.
• Shear-dependent adhesion and biomechanics are abstracted into simplified feedback mechanisms (e.g., shear-dependent inhibition), rather than being explicitly modeled.

The Trpm7 KO simulations aim to demonstrate network robustness rather than exact quantitative agreement with experimental data (e.g., Gotru *et al*. [51]). Our qualitative model of the Boolean network and decision processes deduces the dynamics of simplified interpolation between ON and OFF states. For example, “irreversible aggregation” refers to the committed decision behind aggregation and not a specific measurement or observation of platelet aggregation. The omics data integration heatmap recognizes patterns within each dataset and does not allow cross-dataset quantitative comparison. However, as an additional analysis, we have also compared the log_2_FCs of the platelet CC nodes across a broader panel of disease datasets (17 in total, including multiple cancer types, COVID-19, sepsis, and cardiovascular conditions) by *z* scoring the log_2_FCs across datasets. This cross-dataset comparison is provided in Fig. S16. Because log_2_FCs are already on a comparable scale representing the magnitude of disease-versus-control difference rather than raw platform-specific intensities, this approach is more suitable for cross-dataset comparisons and reveals which CC nodes are consistently dysregulated across disease contexts. However, we retain the *z*-scored intensity/count heatmap in the main figure as it better captures within-dataset dynamic structure, particularly for the phosphor-proteomics time course data where the temporal pattern across stimulation time points is the primary biological signal. Specific further limitations include:

• Batch effects and cross-platform variability: We acknowledge that no batch correction was applied across datasets, which is intentional given that the datasets serve illustrative rather than integrative statistical purposes. The independent *z* scoring mitigates scale differences but cannot correct for platform-specific biases between mass spectrometry and RNA-sequencing.
• RNA–protein discordance and platelet RNA interpretation: Phosphor-proteomics and gene expression data each offer unique insights into platelet biology; however, both come with specific limitations and challenges. Phosphor-proteomics provides a direct view into kinase activity and signaling pathways, which are crucial in platelet function. However, this type of data is often noisy and has high variability due to instability of phosphopeptides and most of the detected phosphopeptides are not functional. Also, the phosphorylation changes are mostly subtle and dynamic. Our pipeline effectively addresses these issues, filtering out the noise to reveal meaningful patterns in platelet signaling. Interpreting gene expression data in platelets is challenging due to their lack of a nucleus. Although platelets cannot transcribe new genes, they have mRNAs from megakaryocytes (MKs), and they can alter their transcriptome through mRNA splicing. This means that in most differential gene expression (DEG) analyses in platelets, we are most likely to capture transcriptomic alterations originating in MKs. This is relevant and useful in infection contexts, where MK transcriptome changes, influenced by the immune response, directly impact platelet function, which is observed in both COVID-19 and sepsis. Integrating several levels of biological information can make the interpretation of the results challenging. There is also a debate within the literature as to how translationally active platelets are, and this could impact the model. However, especially in the case of gene expression, it is best to treat it as a constraint, i.e., if a gene is not expressed, the associated protein cannott participate in signaling interactions.
• Disease-specific confounding: Each transcriptomics dataset reflects a distinct pathological state with its own confounders (medication use, disease severity, comorbidities). The multi-dataset approach in the supplementary log_2_FC heatmap partially addresses this by showing which findings replicate across independent cohorts and disease types, lending greater confidence to nodes that show consistent directionality.

### Implications: First insights of the platelet decision-making model in hemostasis and thrombo-inflammation

The model starts with activating stimuli detected by platelet surface receptors. These receptors then transmit the signal to key proteins like PLC and PI3K, leading to changes in intracellular ion levels, mainly Ca^2+^, but also Zn^2+^ and Mg^2+^. The signal is transduced further and leads to the activation of additional proteins, ultimately causing platelet shape change and platelet activation. Platelets express the integrin αIIbβ3 on their surface. The integrins are activated once platelets are activated. This allows the integrins to stick to each other and form aggregates.

This system ensures that platelets respond to stimuli like injury quickly and stop bleeding as efficiently as possible. The process is carefully regulated to achieve 2 main goals: keeping platelets inactive when there is no injury, and stopping platelet aggregation beyond the sealing of wounds. The first goal is maintained by ECs, which release PGI2 and NO to keep platelets inhibited under normal conditions. The second goal is achieved through both external control by the restored healthy endothelial layer and internal feedback from the platelets, which respond to the increasing shear stress on the growing thrombus. This dual regulation is essential to prevent excessive platelet activation and to ensure that hemostasis is achieved without leading to thrombosis.

The feedback loop plays a crucial role in stabilizing this platelet decision process on the required outcome (here: platelet aggregation) by amplifying the platelet activation signal. This robustness helps minimize the impact of single errors or mutations in signaling proteins, ensuring that hemostasis—a vital mechanism for survival—remains functional and unaffected. In the absence of signal amplification, such mistakes could have a more significant impact. On the other hand, under chronic stress conditions, the same robustness and rapid decision-making that helps with rapid platelet response may contribute to thrombo-inflammation. This occurs when platelets are repeatedly activated by persistent stimuli, such as diabetes, inflammation, infection, aging, or cancer.

Our simulations demonstrate that in the continuous presence of procoagulant stimuli or absence of EC control, platelets can become trapped in a recurring activation loop. The best example of continuous procoagulant stimuli is chronic inflammation. This predicted behavior contrasts sharply with platelets’ role in acute inflammation, where platelet activation serves distinct purposes depending on the stage of the inflammatory response. In the early phases of acute inflammation, such as in response to bacterial or viral infection, platelet activation and coagulation help contain the pathogen at the site of infection, partly by inducing neutrophil NETosis [28]. As inflammation progresses, platelets shift roles, inhibiting neutrophils and suppressing TNF-α secretion from monocytes [89], which resolves the inflammation. The platelet receptor GPIb is central to both the pro- and anti-inflammatory actions of platelets.

However, under conditions of chronic inflammation or persistent oxidative stress, this transition from a pro-inflammatory to an anti-inflammatory state is disrupted, in both platelets and ECs [89]. This disruption increases inflammation and is associated with an increased risk of thrombosis [28]. The failure to transition results in an inflammatory environment that no longer favors resolution of the inflammation but instead promotes ongoing platelet activation and thrombus formation—in line with our simplified platelet decision model.

### Platelet hyperfunction versus hypofunction: Which one is more common?

Although inherited platelet disorders tip the balance to hypofunctional platelets, they are rare and vary from person to person in the clinical outcome [90]. A recent study showed a 3:10^4^ prevalence for clinically relevant loss-of-function variants in platelet genes [91]. On the other hand, thrombosis, caused by hyperfunctioning of platelets, has been described as a major cause of death in the world, accounting for 25% of the deaths worldwide in 2010 [92]. Thrombosis is the top cause of cardiovascular disorders like ischemic heart disease, stroke, and venous thromboembolism (VTE) [93]. Among the 42.0 million deaths from noncommunicable diseases in 2019, ischemic heart disease caused 9.14 million and stroke caused 3.29 million deaths, accounting for 29.5% of deaths worldwide [94]. Additionally, VTE was shown to have a yearly incidence rate of 1 to 2 per 1,000 in Europe and the United States [95]. All combined account for a higher incidence rate than hypofunctioning platelet disorders, and there are far higher numbers of thrombotic events in addition to the deaths caused by the diseases listed, which are already treated and are not included in the incidence rates. In summary, the hyperfunctioning of platelets is more common than hypofunctioning, and this is explained by how platelet signaling is controlled: The system focuses on fast and cascading activation of platelet signaling in the presence of external stimuli. This is in line with the weak effect of specific gene KOs on our platelet decision model, except the receptors (Fig. 8), in the presence of an external stimulus, e.g., damage to the endothelium. On the other hand, according to our model of the platelet decision network, if we have a continuous stimulus or hyperactivation of a platelet protein, the platelet activation status can be irreversibly sustained at its maximum. This is what may happen in pathologies involving hyperactivation of platelets, in which the sustained external stimulus or inhibited external EC control leads to aberrant activation of platelets. The second half of the cycle is closed by platelets activating the cause of their activation—be it inflammation or cancer, bleeding wound closure, or stroke.

### Outlook: Improving translational insights from platelet simulations by means of spatiotemporal agent-based models

There are of course severe translational limits of extrapolating from a single-platelet signaling model to thrombo-inflammatory disease states. Thus, in reality, many individual platelets interact with various other components, in particular the endothel. For this specific shortcoming, spatiotemporal computational simulations allow us to visualize our 7-layered platelet decision model after transfer into a multi-agent environment. To achieve this, our 7-layered signaling network is translated in its key properties into an agent-based model (ABM) [96–98], where one agent represents one platelet and the environment can be the blood vessel—including, in further steps, ECs to study adherence and thrombus formation, or neuronal tissue to study stroke. Box S2 illustrates how we tackle the detailed steps required for this, from the internal life of the individual agent to the fully working spatiotemporal multi-agent simulations: (a) platelet vessel flow, (b) platelet activation, and (c) platelet cell interaction with endothelium including thrombus formation, packing of the platelets within the thrombus in spatiotemporal resolution, and comparison of the simulations to one’s own intravital microscopy images. We want to study later how fast inflammatory changes occur during a stroke [92–94] before and after the thrombus in the brain tissue, and how this can be potentially pharmacologically modulated [99]. For a realistic ABM model, a lot of data are needed, in particular *in vivo* imaging data such as our ongoing study of how platelet glycoprotein V spatiotemporally controls fibrin formation. Moreover, for translational application, we need large-scale data for such specific interventions, for instance, regarding anti-inflammatory treatment in stroke [100]. Finally, and even more time-consuming, clinical studies on the most effective treatments have to pave the way for the clinic.

## Conclusions

We present a 7-layered Boolean model of platelet decision processes that is validated by detailed datasets on platelet signaling and testing of involved key components by mouse gene KOs. Our *in silico* model collates all these different data on platelet signaling, inhibition, and activation in a unified systems-biology model of the involved protein decision network, which is now available to model platelet responses to a range of external and internal stimuli tested, including complex environmental situations such as stroke in the animal model. Limitations include delivery of semiquantitative predictions, no complete biological coverage but rather simplifications, lack of prospective validation, and dependence on literature-curated interactions. As an advantage, the model interpolates signaling behavior for all platelet proteins of this decision-process protein network over time, without being a high resolution and detailed kinetic model of the platelet processes. The model is made fully available and helps to study drug effects on the platelet decision protein network, explore heuristically new and standard pharmacological interventions, and model their resulting effects on platelet signaling.

## Materials

The materials used in this study are shown in Table 1.

**Table 1.** Key resources table: Software and algorithms used for network reconstruction, dynamic simulation, and data analysis, with source references and identifiers (version numbers and URLs)

Resource	Source	Identifier
**Softwares and algorithms**		
dplyr 1.1.4	[105]	https://dplyr.tidyverse.org/
ggplot2 3.5.1	[106]	https://CRAN.R-project.org/package=ggplot2
Jimena	[54,101]	https://www.biozentrum.uni-wuerzburg.de/bioinfo/computing/jimenae/
limma 3.58.1	[107]	https://bioconductor.org/packages/release/bioc/html/limma.html
pheatmap 1.0.12	[108]	https://CRAN.R-project.org/package=pheatmap
PhosR 1.12.0	[109]	https://github.com/PYangLab/PhosR
R 4.3.0 (2023-04-21)—“Already Tomorrow”	N/A	https://cran.r-project.org/
RStudio 2024.04.2+764 (2024.04.2+764)	[110]	https://posit.co/download/rstudio-desktop/
tidyverse 2.0.0	[111]	https://cran.r-project.org/web/packages/tidyverse/index.html
yED Graph Editor	N/A	https://www.yworks.com/products/yed

## Methods

### Network reconstruction and dynamic simulations

We extended our previous model of the platelet central cascade [46,48,50] to include Ca^2+^ and Mg^2+^ transporters, cytoskeletal changes, and environmental feedbacks, and we represented the biological context with positive and negative feedbacks. The constructed model is supported by available literature (Table S1). The interactions were classified either as activating (+) or as inhibiting (−). We constructed and visualized the network topology in the yEd graph editor. We used Jimena [54,101] for semiquantitative simulations of platelet activation using the SQUAD method [53]. JimenaE implements the normalized Hill-function interpolation framework (SQUAD model) to propagate continuous node activity values—ranging from 0 (fully inactive) to 1 (fully active)—at each time step (dt = 0.1).

The network (76 nodes, 115 interactions) is built as a signed directed interaction graph—the foundation of Boolean-style network modeling—in which each edge represents a literature-curated, experimentally supported activation or inhibition interaction. This approach is chosen deliberately: Exact kinetic parameters are unavailable for most interactions at network scale, explicit Boolean gate types (AND, OR, NAND) are rarely resolvable from literature that describes protein interactions only as activation or inhibition, and pure Boolean updating is too coarse to capture graded platelet responses and the temporal order of signaling events. Dynamic simulations are therefore performed using the SQUAD method implemented in Jimena, which translates this interaction topology into continuous ordinary differential equations via normalized Hill functions, yielding node activity values between 0 and 1 and approximating Boolean switching behavior with smooth, biologically meaningful dynamics without requiring full kinetic parameterization.

Prior to dynamic simulations, the quiescent steady state was identified using JimenaE’s built-in exhaustive steady-state search; all simulations were initialized from this state, in which healthy EC signaling is active and the damage/injury input is set to 0. Perturbations were introduced by clamping individual node values: The TRPM7 kinase domain was set to 1 (WT) or 0 (KO); disease states were modeled by incrementally increasing disease-specific input nodes (atherosclerosis, IA, TNF-α) while adjusting the healthy EC layer; genetic disorders were modeled by setting the relevant receptor node (GPVI, αIIbβ3, or GPIb-IX-V) to 0. We also reproduced several experimental and pathological conditions by introducing perturbations to our *in silico* simulations. Details for each simulation can be found in Table S3.

The model parametrization is automatically done by the software Jimena [54] according to the Boolean network supplied.

For the same network and its logical connectivity, you get always the same response behavior if you use the same version of the interpolation program Jimena. Apart from specific perturbation scenarios (see paragraph above), there were no additional node constraints, just the Boolean connectivity was always respected. Activation and inhibition of specific nodes influence of course the dynamic behavior This is important in particular for the disease input nodes and explained in Results (Fig. S9), taking atherosclerosis as example; for gene KOs, we assumed simply that the node is 100% inhibited. Otherwise, we used the default parameters throughout (see [54]) regarding Hill function and interpolation and convergence/time step settings.

### Integrating platelet data from different diseases

We analyzed gene-expression and phosphorylation changes in platelet central cascade (CC) nodes across 4 datasets:

1. ACKR3 dataset 1, a phosphor-proteomics dataset with 7 time points following ACKR3 stimulation;
2. ACKR3 dataset 2, a validation phosphor-proteomics dataset with 3 main time points and DMSO controls for each time point;
3. the Cancer dataset, an RNA-sequencing dataset from non-small cell lung cancer (NSCLC) patients. This tumor-educated platelet gene expression dataset was originally created to study swarm-intelligence-enhanced detection of lung cancer cells [102] and is available from the Gene Expression Omnibus (GEO) as original dataset GSE89843.
4. the COVID-19 dataset, an RNA-sequencing dataset from COVID-19 patients. Publicly available sequencing datasets were analyzed in [44]. The original data can be found at the following bioproject of the NIH: https://www.ncbi.nlm.nih.gov/bioproject/PRJNA634489/ [103].

All 4 datasets are given in Table S2.

The raw data for ACKR3 dataset 1 and dataset 2 are deposited with PRIDE (PXD074638, doi:10.6019/PXD074638).

For each dataset, we included all relevant platelet CC node genes, encompassing isoforms and subunits where applicable. For ACKR3 dataset 1, we selected one phosphorylation site per protein. The selected site was the one that demonstrated the most significant change across the time points. To determine this, we performed an analysis of variance (ANOVA) test on the phosphorylation data for each protein and identified the site with the lowest *P* value.

In ACKR3 dataset 2, we calculated the ACKR3-minus-DMSO differences to focus on the changes induced by ACKR3 stimulation specifically and performed an ANOVA test on these values. From these results, we selected the phosphorylation site for each protein that exhibited the highest variation in these differences over time. The site with the lowest *P* value from the ANOVA test was chosen as the most significantly changing site.

To enable within-dataset analysis, we normalized the counts and intensities from each dataset independently and transformed the data into *z* scores. As each dataset underwent separate normalization and transformation, direct comparisons of absolute expression levels between datasets were not performed.

### Platelet sample preparation and phosphor-proteomics (ACKR3 datasets)

The phosphor-proteomics experiments underlying ACKR3 datasets 1 and 2 are described in full detail in the companion manuscript (Balkenhol *et al*., in preparation: “Atypical chemokine receptor ACKR3/CXCR7 modulated platelet phosphoproteome drives the anti-thrombotic effects”). Briefly, washed platelets from healthy donors (dataset 1: *n* = 8, 7 time points post-ACKR3-agonist stimulation at 100 μg/ml; dataset 2: *n* = 10, 3 time points with DMSO controls) were lysed, reduced, alkylated, and digested by trypsin using the filter-aided sample preparation (FASP) protocol. Phosphopeptides were enriched using Fe(III)-NTA IMAC resin (Agilent) on an automated Bravo AssayMAP platform. Liquid chromatography–tandem mass spectrometry (LC-MS/MS) was performed on a U3000 RSLCnano ProFlow system coupled to a Q Exactive HF mass spectrometer (Thermo Scientific) in data-dependent acquisition (DDA) mode. Raw files were processed with Proteome Discoverer 2.5 using Mascot 2.6 [false discovery rate (FDR) 1% at peptide spectrum matching (PSM)/peptide level] and phosphosite localization by ptmRS. Statistical preprocessing included log₂ transformation, median normalization, protein-level normalization of phosphopeptide abundances, and donor-effect correction using RUVphospho (*k* = 16) with 479 (dataset 1) or 380 (dataset 2) empirically derived control phosphopeptides. Differential-abundance analysis used the limma package (voom/lmFit/eBayes, Benjamini–Hochberg FDR correction); sites with adjusted *P* value < 0.05 and |log₂FC| > 0.5 were considered significant.

### Bioinformatic omics data analysis

For the transcriptomics datasets, the baseline is the healthy control group from each respective study, against which disease samples were compared in the differential expression analysis. *Z* scores were then calculated row-wise across the condition averages within each dataset. For the phosphor-proteomics datasets, baseline corresponds to the unstimulated condition (*t*_0_), with *z* scores calculated across time points per protein per dataset.

The full analysis scripts and raw mass-spectrometry data for the ACKR3 phosphor-proteomics datasets will be made publicly available upon publication of the companion manuscript (Balkenhol *et al*., in preparation: “Atypical chemokine receptor ACKR3/CXCR7 modulated platelet phosphoproteome drives the anti-thrombotic effects”). The data have been deposited in ProteomeXchange via PRIDE (PXD074638, doi:10.6019/PXD074638) [104] and will be released upon acceptance of the companion paper. Reviewer access is available upon request to the corresponding author.

## Data Availability

Omics datasets. The processed omics data used for the 4-dataset integration are provided in Table S2. Raw mass-spectrometry phosphor-proteomics data (ACKR3 datasets 1 and 2) are deposited in ProteomeXchange via PRIDE (identifier PXD074638, doi:10.6019/PXD074638) [104]. The TEP transcriptomics dataset is deposited at NCBI GEO (accession GSE89843). The COVID-19 transcriptomics dataset is deposited at NCBI SRA (accession PRJNA634489). Platelet signaling network and simulation files. To ensure full reproducibility and to support community reuse, the following resources are deposited in a Zenodo repository supporting the publication of this manuscript: (a) the complete 7-layer platelet signaling network topology in GraphML format, compatible with yED Graph Editor (network files for Figs. 1 to 3 and File S4); (b) the literature-curated edge table (Table S1) listing all 115 interactions with their supporting literature references, activation/inhibition type, and layer assignment, providing full transparency of how the network was constructed; (c) Jimena simulation project files allowing direct reproduction of all simulations presented in this manuscript; and (d) the analysis scripts for the omics data integration. The title and link is: johannes-balkenhol. (2026). johannes-balkenhol/seven-layer-platelet-signaling-network: v1.0—Initial release for CSBJ submission (version v1.0) [Computer software]. Zenodo. https://doi.org/10.5281/zenodo.22079746. Jimena software is freely available at https://www.biozentrum.uni-wuerzburg.de/bioinfo/computing/jimena2/.

## Supplementary Materials

Supplementary 1 Supplementary Text Figs. S1 to S16 Tables S1 to S3 Boxes S1 and S2 Files S1 to S4

## References

[1] Assinger A, Kral JB, Yaiw KC, Schrottmaier WC, Kurzejamska E, Wang Y, Mohammad AA, Religa P, Rahbar A, Söderberg-Nauclér C, et al. Human cytomegalovirus–platelet interaction triggers toll-like receptor 2–dependent proinflammatory and proangiogenic responses. Arterioscler Thromb Vasc Biol. 2014;34(4):801–809. 10.1161/ATVBAHA.114.303287 24558109

[2] McMullen PD, Cho JH, Miller JL, Husain AN, Pytel P, Krausz T. A descriptive and quantitative immunohistochemical study demonstrating a spectrum of platelet recruitment patterns across pulmonary infections including COVID-19. Am J Clin Pathol. 2021;155(3):354–363. 10.1093/ajcp/aqaa230 PMC7717231 33174599

[3] Zhang S, Liu Y, Wang X, Yang L, Li H, Wang Y, Liu M, Zhao X, Xie Y, Yang Y, et al. SARS-CoV-2 binds platelet ACE2 to enhance thrombosis in COVID-19. J Hematol Oncol. 2020a;13(1):120. 10.1186/s13045-020-00954-7 PMC7471641 32887634

[4] Zhang Y, Zeng X, Jiao Y, Li Z, Liu Q, Ye J, Yang M. Mechanisms involved in the development of thrombocytopenia in patients with COVID-19. Thromb Res. 2020b;193:110–115. 10.1016/j.thromres.2020.06.008 PMC7274097 32535232

[5] Boilard E, Nigrovic PA, Larabee K, Watts GFM, Coblyn JS, Weinblatt ME, Massarotti EM, Remold-O’Donnell E, Farndale RW, Ware J, et al. Platelets amplify inflammation in arthritis via collagen-dependent microparticle production. Science. 2010;327(5965):580–583. 10.1126/science.1181928 PMC2927861 20110505

[6] Golebiewska EM, Poole AW. Platelet secretion: From haemostasis to wound healing and beyond. Blood Rev. 2015;29(3):153–162. 10.1016/j.blre.2014.10.003 PMC4452143 25468720

[7] Coenen DM, Mastenbroek TG, Cosemans JMEM. Platelet interaction with activated endothelium: Mechanistic insights from microfluidics. Blood. 2017;130(26):2819–2828. 10.1182/blood-2017-04-780825 29018081

[8] Finsterbusch M, Schrottmaier WC, Kral-Pointner JB, Salzmann M, Assinger A. Measuring and interpreting platelet-leukocyte aggregates. Platelets. 2018;29(7):677–685. 10.1080/09537104.2018.1430358 PMC6178087 29461910

[9] Hottz ED, Azevedo-Quintanilha IG, Palhinha L, Teixeira L, Barreto EA, Pao CRR, Righy C, Franco S, Souza TML, Kurtz P, et al. Platelet activation and platelet-monocyte aggregate formation trigger tissue factor expression in patients with severe COVID-19. Blood. 2020;136(11):1330–1341. 10.1182/blood.2020007252 PMC7483437 32678428

[10] Lisman T. Platelet-neutrophil interactions as drivers of inflammatory and thrombotic disease. Cell Tissue Res. 2018;371(3):567–576. 10.1007/s00441-017-2727-4 PMC5820397 29178039

[11] Mussbacher M, Pirabe A, Brunnthaler L, Schrottmaier WC, Assinger A. Horizontal microRNA transfer by platelets—Evidence and implications. Front Physiol. 2021;12: Article 678362. 10.3389/fphys.2021.678362 PMC8209332 34149456

[12] Schrottmaier WC, Mussbacher M, Salzmann M, Assinger A. Platelet–leukocyte interplay during vascular disease. Atherosclerosis. 2020;307:109–120. 10.1016/j.atherosclerosis.2020.04.018 32439204

[13] Nardi AC, Coy-Canguçu A, Saito A, Florio MF, Marti G, Degasperi GR, Orsi FA. Immunothrombosis and its underlying biological mechanisms. Hematol Transfus Cell Ther. 2024;46(1):49–57. 10.1016/j.htct.2023.05.008 PMC10935458 37451977

[14] Guo L, Rondina MT. The era of thromboinflammation: Platelets are dynamic sensors and effector cells during infectious diseases. Front Immunol. 2019;10:2204. 10.3389/fimmu.2019.02204 PMC6753373 31572400

[15] Williams MR, Azcutia V, Newton G, Alcaide P, Luscinskas FW. Emerging mechanisms of neutrophil recruitment across endothelium. Trends Immunol. 2011;32(10):461–469. 10.1016/j.it.2011.06.009 PMC3185121 21839681

[16] Anvari S, Osei E, Maftoon N. Interactions of platelets with circulating tumor cells contribute to cancer metastasis. Sci Rep. 2021;11(1):15477. 10.1038/s41598-021-94735-y PMC8322323 34326373

[17] Li Y, Wang H, Zhao Z, Yang Y, Meng Z, Qin L. Effects of the interactions between platelets with other cells in tumor growth and progression. Front Immunol. 2023;14:1165989. 10.3389/fimmu.2023.1165989 PMC10158495 37153586

[18] Suzuki-Inoue K. Platelets and cancer-associated thrombosis: Focusing on the platelet activation receptor CLEC-2 and podoplanin. Hematology Am Soc Hematol Educ Program. 2019;2019(1):175–181. 10.1182/hematology.2019001388 PMC6913448 31808911

[19] Yu L, Guo Y, Chang Z, Zhang D, Zhang S, Pei H, Pang J, Zhao ZJ, Chen Y. Bidirectional interaction between cancer cells and platelets provides potential strategies for cancer therapies. Front Oncol. 2021;11: Article 764119. 10.3389/fonc.2021.764119 PMC8551800 34722319

[20] Jackson SP, Darbousset R, Schoenwaelder SM. Thromboinflammation: Challenges of therapeutically targeting coagulation and other host defense mechanisms. Blood. 2019;133(9):906–918. 10.1182/blood-2018-11-882993 30642917

[21] Mack A, Vanden Hoek T, Du X. Thromboinflammation and the role of platelets. Arterioscler Thromb Vasc Biol. 2024;44(6):1175–1180. 10.1161/ATVBAHA.124.320149 PMC11649024 38776384

[22] van der Meijden PEJ, Heemskerk JWM. Platelet biology and functions: New concepts and clinical perspectives. Nat Rev Cardiol. 2019;16(3):166–179. 10.1038/s41569-018-0110-0 30429532

[23] Wagner DD, Heger LA. Thromboinflammation: From atherosclerosis to COVID-19. Arterioscler Thromb Vasc Biol. 2022;42(9):1103–1112. 10.1161/ATVBAHA.122.317162 PMC9420806 35861953

[24] Klavina PA, Leon G, Curtis AM, Preston RJS. Dysregulated haemostasis in thrombo-inflammatory disease. Clin Sci. 2022;136(24):1809–1829. 10.1042/CS20220208 PMC9760580 36524413

[25] Sharma S, Tyagi T, Antoniak S. Platelet in thrombo-inflammation: Unraveling new therapeutic targets. Front Immunol. 2022;13:1039843. 10.3389/fimmu.2022.1039843 PMC9702553 36451834

[26] Chen Y, Zhong H, Zhao Y, Luo X, Gao W. Role of platelet biomarkers in inflammatory response. Biomark Res. 2020;8:28. 10.1186/s40364-020-00207-2 PMC7397646 32774856

[27] Martinez Bravo G, Annarapu G, Carmona E, Nawarskas J, Clark R, Novelli E, Mota Alvidrez RI. Platelets in thrombosis and atherosclerosis: A double-edged sword. Am J Pathol. 2024;194(9):1608–1621. 10.1016/j.ajpath.2024.05.010 PMC11373056 38885926

[28] Stark K, Massberg S. Interplay between inflammation and thrombosis in cardiovascular pathology. Nat Rev Cardiol. 2021;18(9):666–682. 10.1038/s41569-021-00552-1 PMC8100938 33958774

[29] Jamasbi J, Megens RTA, Bianchini M, Münch G, Ungerer M, Faussner A, Sherman S, Walker A, Goyal P, Jung S, et al. Differential inhibition of human atherosclerotic plaque-induced platelet activation by dimeric GPVI-Fc and anti-GPVI antibodies: Functional and imaging studies. J Am Coll Cardiol. 2015;65(22):2404–2415. 10.1016/j.jacc.2015.03.573 PMC4452546 26046734

[30] Walsh TG, Berndt MC, Carrim N, Cowman J, Kenny D, Metharom P. The role of Nox1 and Nox2 in GPVI-dependent platelet activation and thrombus formation. Redox Biol. 2014;2:178–186. 10.1016/j.redox.2013.12.023 PMC3909778 24494191

[31] Yong J, Toh C-H. The convergent model of coagulation. J Thromb Haemost. 2024;22(8):2140–2146. 10.1016/j.jtha.2024.05.014 38815754

[32] Davi G, Patrono C. Platelet activation and atherothrombosis. N Engl J Med. 2007;357(24):2482–2494. 10.1056/NEJMra071014 18077812

[33] Mac Mullan PA, Peace AJ, Madigan AM, Tedesco AF, Kenny D, McCarthy GM. Platelet hyper-reactivity in active inflammatory arthritis is unique to the adenosine diphosphate pathway: A novel finding and potential therapeutic target. Rheumatology. 2010;49(2):240–245. 10.1093/rheumatology/kep377 19965976

[34] Gu SX, Dayal S. Inflammation-mediated platelet hyperactivity in aging. Ann Blood. 2020;5:10. 10.21037/aob.2020.03.01 PMC7286603 32524077

[35] Price J, Lord JM, Harrison P. Inflammaging and platelet hyperreactivity: A new therapeutic target? J Thromb Haemost. 2020;18(1):3–5. 10.1111/jth.14670 31894663

[36] Davizon-Castillo P, McMahon B, Aguila S, Bark D, Ashworth K, Allawzi A, Campbell RA, Montenont E, Nemkov T, D’Alessandro A, et al. TNF-α-driven inflammation and mitochondrial dysfunction define the platelet hyperreactivity of aging. Blood. 2019;134(9):727–740. 10.1182/blood.2019000200 PMC6716075 31311815

[37] Cognasse F, Nguyen KA, Heestermans M, Arthaud CA, Eyraud MA, Prier A, De Bernard S, Nourikyan J, Duchez AC, Avril S, et al. Computational modeling of platelet activation signatures in response to diverse immune and hemostatic agonists. Platelets. 2025;36(1):2572982. 10.1080/09537104.2025.2572982 41143469

[38] van Holten TC, Bleijerveld OB, Wijten P, de Groot PG, Heck AJ, Barendrecht AD, Merkx TH, Scholten A, Roest M. Quantitative proteomics analysis reveals similar release profiles following specific PAR-1 or PAR-4 stimulation of platelets. Cardiovasc Res. 2014;103(1):140–146. 10.1093/cvr/cvu113 24776597

[39] Kamykowski J, Carlton P, Sehgal S, Storrie B. Quantitative immunofluorescence mapping reveals little functional coclustering of proteins within platelet alpha-granules. Blood. 2011;118(5):1370–1373. 10.1182/blood-2011-01-330910 21622648

[40] Zhou Y, Yasumoto A, Lei C, Huang CJ, Kobayashi H, Wu Y, Yan S, Sun CW, Yatomi Y, Goda K. Intelligent classification of platelet aggregates by agonist type. Elife. 2020;9: Article e52938. 10.7554/eLife.52938 PMC7217700 32393438

[41] Gerda B, Volkova A, Dobrylko I, Andreyeva AY, Dandekar T, Panteleev MA, Gambaryan S, Mindukshev I. Experimental and mathematical model of platelet hemostasis kinetics. Cells. 2025;14(9):677. 10.3390/cells14090677 PMC12071887 40358201

[42] Breitenbach T, Englert N, Osmanoglu Ö, Rukoyatkina N, Wangorsch G, Heinze K, Friebe A, Butt E, Feil R, Dittrich M, et al. A modular systems-biological modelling framework studies cyclic nucleotide signaling in platelets. J Theor Biol. 2022;550: Article 111222. 10.1016/j.jtbi.2022.111222 35843440

[43] Wangorsch G, Butt E, Mark R, Hubertus K, Geiger J, Dandekar T, Dittrich M. Time-resolved in silico modeling of fine-tuned cAMP signaling in platelets: Feedback loops, titrated phosphorylations and pharmacological modulation. BMC Syst Biol. 2011;5(1):178. 10.1186/1752-0509-5-178 PMC3247139 22034949

[44] Osmanoglu Ö, Gupta SK, Almasi A, Yagci S, Srivastava M, Araujo GH, Nagy Z, Balkenhol J, Dandekar T. Signaling network analysis reveals fostamatinib as a potential drug to control platelet hyperactivation during SARS-CoV-2 infection. Front Immunol. 2023;14:1285345. 10.3389/fimmu.2023.1285345 PMC10768010 38187394

[45] Osmanoglu Ö, Khan MMT, Karl S, Bhattacharyya M, Dittrich M, Dandekar T. Network controllability reveals key mitigation points for tumor-promoting signaling in tumor-educated platelets. Int J Mol Sci. 2025;26(21):10780. 10.3390/ijms262110780 PMC12609506 41226816

[46] Balkenhol J, Kaltdorf KV, Mammadova-Bach E, Braun A, Nieswandt B, Dittrich M, Dandekar T. Comparison of the central human and mouse platelet signaling cascade by systems biological analysis. BMC Genomics. 2020;21(1):897. 10.1186/s12864-020-07215-4 PMC7756956 33353544

[47] Boyanova D, Nilla S, Birschmann I, Dandekar T, Dittrich M. PlateletWeb: A systems biologic analysis of signaling networks in human platelets. Blood. 2012;119(3):e22–e34. 10.1182/blood-2011-10-387308 22123846

[48] Mischnik M, Boyanova D, Hubertus K, Geiger J, Philippi N, Dittrich M, Wangorsch G, Timmer J, Dandekar T. A Boolean view separates platelet activatory and inhibitory signalling as verified by phosphorylation monitoring including threshold behaviour and integrin modulation. Mol BioSyst. 2013a;9(6):1326–1339. 10.1039/c3mb25597b 23463387

[49] Mischnik M, Hubertus K, Geiger J, Dandekar T, Timmer J. Dynamical modelling of prostaglandin signalling in platelets reveals individual receptor contributions and feedback properties. Mol BioSyst. 2013b;9(10):2520–2529. 10.1039/c3mb70142e 23903629

[50] Mischnik M, Gambaryan S, Subramanian H, Geiger J, Schütz C, Timmer J, Dandekar T. A comparative analysis of the bistability switch for platelet aggregation by logic-ODE based dynamical modeling. Mol BioSyst. 2014;10(8):2082–2089. 10.1039/c4mb00170b 24852796

[51] Gotru SK, Chen W, Kraft P, Becker IC, Wolf K, Stritt S, Zierler S, Hermanns HM, Rao D, Perraud A-L, et al. TRPM7 kinase controls calcium responses in arterial thrombosis and stroke in mice. Arterioscler Thromb Vasc Biol. 2018;38(2):344–352. 10.1161/ATVBAHA.117.310391 29146750

[52] Czakai K, Dittrich M, Kaltdorf M, Müller T, Krappmann S, Schedler A, Bonin M, Dühring S, Schuster S, Speth C, et al. Influence of platelet-rich plasma on the immune response of human monocyte-derived dendritic cells and macrophages stimulated with Aspergillus fumigatus. Int J Med Microbiol. 2017;307(2):95–107. 10.1016/j.ijmm.2016.11.010 27965080

[53] Di Cara A, Garg A, De Micheli G, Xenarios I, Mendoza L. Dynamic simulation of regulatory networks using SQUAD. BMC Bioinformatics. 2007;8:462. 10.1186/1471-2105-8-462 PMC2238325 18039375

[54] Kaltdorf M, Breitenbach T, Karl S, Fuchs M, Kessie DK, Psota E, Prelog M, Sarukhanyan E, Ebert R, Jakob F, et al. Software JimenaE allows efficient dynamic simulations of Boolean networks, centrality and system state analysis. Sci Rep. 2023;13(1):1855. 10.1038/s41598-022-27098-7 PMC9892028 36725967

[55] Shehwar D, Barki S, Aliotta A, Calderara DB, Veuthey L, Portela CP, Alberio L, Alam MR. Platelets and mitochondria: The calcium connection. Mol Biol Rep. 2025;52(1):276. 10.1007/s11033-025-10389-3 40029418

[56] Damiano-Guercio J, Kurzawa L, Mueller J, Dimchev G, Schaks M, Nemethova M, Pokrant T, Brühmann S, Linkner J, Blanchoin L, et al. Loss of Ena/VASP interferes with lamellipodium architecture, motility and integrin-dependent adhesion. Elife. 2020;9: Article e55351. 10.7554/eLife.55351 PMC7239657 32391788

[57] Gachet C, Hechler B. Platelet purinergic receptors in thrombosis and inflammation. Hamostaseologie. 2020;40(2):145–152. 10.1055/a-1113-0711 32464678

[58] Tokarz-Deptuła B, Frączyk M, Czupryńska P, Stosik M, Deptuła W. Characterization of platelet receptors and their involvement in immune activation of these cells. Int J Mol Sci. 2024;25(23):12611. 10.3390/ijms252312611 PMC11641666 39684330

[59] Swieringa F, Heemskerk JWM, Assinger A. Platelet activation and signaling in thrombus formation. Blood. 2025;146(12):1400–1411. 10.1182/blood.2024025320 39869836

[60] Tian Y, Zong Y, Pang Y, Zheng Z, Ma Y, Zhang C, Gao J. Platelets and diseases: Signal transduction and advances in targeted therapy. Signal Transduct Target Ther. 2025;10(1):159. 10.1038/s41392-025-02198-8 PMC12081703 40374650

[61] Yau JW, Teoh H, Verma S. Endothelial cell control of thrombosis. BMC Cardiovasc Disord. 2015;15:130. 10.1186/s12872-015-0124-z PMC4617895 26481314

[62] Neubauer K, Zieger B. Endothelial cells and coagulation. Cell Tissue Res. 2022;387(3):391–398. 10.1007/s00441-021-03471-2 PMC8975780 34014399

[63] Yeung J, Li W, Holinstat M. Platelet signaling and disease: Targeted therapy for thrombosis and other related diseases. Pharmacol Rev. 2018;70(3):526–548. 10.1124/pr.117.014530 PMC6013590 29925522

[64] Feske S, Gwack Y, Prakriya M, Srikanth S, Puppel S-H, Tanasa B, Hogan PG, Lewis RS, Daly M, Rao A. A mutation in Orai1 causes immune deficiency by abrogating CRAC channel function. Nature. 2006;441(7090):179–185. 10.1038/nature04702 16582901

[65] Hofmann T, Obukhov AG, Schaefer M, Harteneck C, Gudermann T, Schultz G. Direct activation of human TRPC6 and TRPC3 channels by diacylglycerol. Nature. 1999;397(6716):259–263. 10.1038/16711 9930701

[66] Varga-Szabo D, Braun A, Kleinschnitz C, Bender M, Pleines I, Pham M, Renné T, Stoll G, Nieswandt B. The calcium sensor STIM1 is an essential mediator of arterial thrombosis and ischemic brain infarction. J Exp Med. 2008;205(7):1583–1591. 10.1084/jem.20080302 PMC2442636 18559454

[67] Penner R, Fleig A. The Mg2+ and Mg2+-nucleotide-regulated channel-kinase TRPM7. Handb Exp Pharmacol. 2007;179:313–328. 10.1007/978-3-540-34891-7_19 PMC5663631 17217066

[68] Suzuki S, Lis A, Schmitz C, Penner R, Fleig A. The TRPM7 kinase limits receptor-induced calcium release by regulating heterotrimeric G-proteins. Cell Mol Life Sci. 2018;75(16):3069–3078. 10.1007/s00018-018-2786-z PMC6033657 29500477

[69] Bye AP, Unsworth AJ, Gibbins JM. Platelet signaling: A complex interplay between inhibitory and activatory networks. J Thromb Haemost. 2016;14(5):918–930. 10.1111/jth.13302 PMC4879507 26929147

[70] Sun H, Lagarrigue F, Ginsberg MH. The connection between Rap1 and Talin1 in the activation of integrins in blood cells. Front Cell Dev Biol. 2022;10: Article 908622. 10.3389/fcell.2022.908622 PMC9198492 35721481

[71] Aburima A, Wraith KS, Raslan Z, Law R, Magwenzi S, Naseem KM. cAMP signaling regulates platelet myosin light chain (MLC) phosphorylation and shape change through targeting the RhoA–Rho kinase–MLC phosphatase signaling pathway. Blood. 2013;122(20):3533–3545. 10.1182/blood-2013-03-487850 24100445

[72] Nagy Z, Smolenski A. Cyclic nucleotide-dependent inhibitory signaling interweaves with activating pathways to determine platelet responses. Res Pract Thromb Haemost. 2018;2(3):558–571. 10.1002/rth2.12122 PMC6046581 30046761

[73] Wen L, Feil S, Wolters M, Thunemann M, Regler F, Schmidt K, Friebe A, Olbrich M, Langer H, Gawaz M, et al. A shear-dependent NO-cGMP-cGKI cascade in platelets acts as an auto-regulatory brake of thrombosis. Nat Commun. 2018;9(1):4301. 10.1038/s41467-018-06638-8 PMC6191445 30327468

[74] Burkhart JM, Vaudel M, Gambaryan S, Radau S, Walter U, Martens L, Geiger J, Sickmann A, Zahedi RP. The first comprehensive and quantitative analysis of human platelet protein composition allows the comparative analysis of structural and functional pathways. Blood. 2012;120(15):e73–e82. 10.1182/blood-2012-04-416594 22869793

[75] Zahedi RP, Lewandrowski U, Wiesner J, Wortelkamp S, Moebius J, Schutz C, Walter U, Gambaryan S, Sickmann A. Phosphoproteome of resting human platelets. J Proteome Res. 2008;7(2):526–534. 10.1021/pr0704130 18088087

[76] Beck F, Geiger J, Gambaryan S, Solari FA, Dell’Aica M, Loroch S, Mattheij NJ, Mindukshev I, Pötz O, Jurk K, et al. Temporal quantitative phosphor-proteomics of ADP stimulation reveals novel central nodes in platelet activation and inhibition. Blood. 2017;129(2):e1–e12. 10.1182/blood-2016-05-714048 PMC5248936 28060719

[77] Stalker TJ, Newman DK, Ma P, Wannemacher KM, Brass LF. Platelet signaling. Handb Exp Pharmacol. 2012;210:59–85. 10.1007/978-3-642-29423-5_3 PMC4785017 22918727

[78] Estevez B, Du X. New concepts and mechanisms of platelet activation signaling. Physiology. 2017;32(2):162–177. 10.1152/physiol.00020.2016 PMC5337829 28228483

[79] Golebiewska EM, Poole AW. Secrets of platelet exocytosis—What do we really know about platelet secretion mechanisms? Br J Haematol. 2013;165(2):204–216. 10.1111/bjh.12682 PMC4155865 24588354

[80] Li Z, Delaney MK, O’Brien KA, Du X. Signaling during platelet adhesion and activation. Arterioscler Thromb Vasc Biol. 2010;30(12):2341–2349. 10.1161/ATVBAHA.110.207522 PMC3085271 21071698

[81] Lopez-Vilchez I, Diaz-Ricart M, White JG, Escolar G, Galan AM. Serotonin enhances platelet procoagulant properties and their activation induced during platelet tissue factor uptake. Cardiovasc Res. 2009;84(2):309–316. 10.1093/cvr/cvp205 19541671

[82] Oury C, Lecut C, Hego A, Wera O, Delierneux C. Purinergic control of inflammation and thrombosis: Role of P2X1 receptors. Comput Struct Biotechnol J. 2015;13:106–110. 10.1016/j.csbj.2014.11.008 PMC4334884 25709760

[83] Makhoul S, Walter E, Pagel O, Walter U, Sickmann A, Gambaryan S, Smolenski A, Zahedi RP, Jurk K. Effects of the NO/soluble guanylate cyclase/cGMP system on the functions of human platelets. Nitric Oxide. 2018;76:71–80. 10.1016/j.niox.2018.03.008 29550521

[84] Feil R, Lehners M, Stehle D, Feil S. Visualising and understanding cGMP signals in the cardiovascular system. Br J Pharmacol. 2022;179(11):2394–2412. 10.1111/bph.15500 33880767

[85] Aurbach K, Spindler M, Haining EJ, Bender M, Pleines I. Blood collection, platelet isolation and measurement of platelet count and size in mice—A practical guide. Platelets. 2019;30(6):698–707. 10.1080/09537104.2018.1528345 30346859

[86] Nugent D, Kunicki T. Platelet genomics: The role of platelet size and number in health and disease. Platelets. 2017;28(1):27–33. 10.1080/09537104.2016.1257113 28032522

[87] Palma-Barqueros V, Revilla N, Sanchez A, Zamora Cañaveras A, Rodriguez-Alen A, Marin-Quilez A, Gonzalez-Porras JR, Vicente V, Lozano ML, Bastida JM, et al. Inherited platelet disorders: An updated overview. Int J Mol Sci. 2021;22(9):4521. 10.3390/ijms22094521 PMC8123627 33926054

[88] Berndt MC, Andrews RK. Bernard-Soulier syndrome. Haematologica. 2011;96(3):355–359. 10.3324/haematol.2010.039883 PMC3046265 21357716

[89] Corken A, Russell S, Dent J, Post SR, Ware J. Platelet glycoprotein Ib-IX as a regulator of systemic inflammation. Arterioscler Thromb Vasc Biol. 2014;34(5):996–1001. 10.1161/ATVBAHA.113.303113 PMC3991762 24504734

[90] Bastida JM, González-Porras JR, Jiménez C, Benito R, Ordoñez GR, Alvarez-Román MT, Fisac RM, González-López TJ, Rivera J, Lozano ML, et al. Molecular diagnosis of inherited coagulation and bleeding disorders. Semin Thromb Hemost. 2019;45(7):695–707. 10.1055/s-0039-1687889 31041795

[91] Oved JH, Lambert MP, Kowalska MA, Poncz M, Karczewski KJ. Population-based frequency of naturally occurring loss-of-function variants in genes associated with platelet disorders. J Thromb Haemost. 2021;19(1):248–254. 10.1111/jth.15113 33006441

[92] Wendelboe AM, Raskob GE. Global burden of thrombosis: Epidemiologic aspects. Circ Res. 2016;118(9):1340–1347. 10.1161/CIRCRESAHA.115.306841 27126645

[93] Raskob GE, Angchaisuksiri P, Blanco AN, Büller H, Gallus A, Hunt BJ, Hylek EM, Kakkar A, Konstantinides SV, McCumber M, et al. Thrombosis: A major contributor to global disease burden. Arterioscler Thromb Vasc Biol. 2014;34(11):2363–2371. 10.1161/ATVBAHA.114.304488 25304324

[94] GBD 2019 Diseases and Injuries Collaborators. Global burden of 369 diseases and injuries in 204 countries and territories, 1990–2019: A systematic analysis for the Global Burden of Disease Study 2019. Lancet. 2020;396(10258):1204–1222. 10.1016/S0140-6736(20)30925-9 PMC7567026 33069326

[95] Lutsey PL, Zakai NA. Epidemiology and prevention of venous thromboembolism. Nat Rev Cardiol. 2023;20(4):248–262. 10.1038/s41569-022-00787-6 PMC9579604 36258120

[96] Schneider KM, Giehl K, Baeurle SA. Development and application of an agent-based model for the simulation of the extravasation process of circulating tumor cells. Int J Numer Method Biomed Eng. 2023;39(4): Article e3679. 10.1002/cnm.3679 36606741

[97] Tantiwong C, Dunster JL, Cavill R, Tomlinson MG, Wierling C, Heemskerk JWM, Gibbins JM. An agent-based approach for modelling and simulation of glycoprotein VI receptor diffusion, localisation and dimerisation in platelet lipid rafts. Sci Rep. 2023;13(1):3906. 10.1038/s41598-023-30884-6 PMC9994409 36890261

[98] Uppal A, Wightman SC, Ganai S, Weichselbaum RR, An G. Investigation of the essential role of platelet–tumor cell interactions in metastasis progression using an agent-based model. Theor Biol Med Model. 2014;11(1):17. 10.1186/1742-4682-11-17 PMC4022382 24725600

[99] Stoll G, Nieswandt B, Schuhmann MK. Ischemia/reperfusion injury in acute human and experimental stroke: Focus on thrombo-inflammatory mechanisms and treatments. Neurol Res Pract. 2024;6(1):57. 10.1186/s42466-024-00355-y PMC11587771 39582054

[100] Beck S, Öftering P, Li R, Hemmen K, Nagy M, Wang Y, Zarpellon A, Schuhmann MK, Stoll G, Ruggeri ZM, et al. Platelet glycoprotein V spatio-temporally controls fibrin formation. Nat Cardiovasc Res. 2023;2(4):368–382. 10.1038/s44161-023-00254-6 PMC10195106 37206993

[101] Karl S, Dandekar T. Jimena: Efficient computing and system state identification for genetic regulatory networks. BMC Bioinformatics. 2013;14(1):306. 10.1186/1471-2105-14-306 PMC3853020 24118878

[102] Best MG, Sol N, In’t Veld SGJG, Vancura A, Muller M, Niemeijer ALN, Fejes AV, Kon Fat LAT, In’t Veld AEH, Leurs C, et al. Swarm intelligence-enhanced detection of non-small-cell lung cancer using tumor-educated platelets. Cancer Cell. 2017;32(2):238–252.e9. 10.1016/j.ccell.2017.07.004 PMC6381325 28810146

[103] Manne BK, Denorme F, Middleton EA, Portier I, Rowley JW, Stubben C, Petrey AC, Tolley ND, Guo L, Cody M, et al. Platelet gene expression and function in patients with COVID-19. Blood. 2020;136(11):1317–1329. 10.1182/blood.2020007214 PMC7483430 32573711

[104] Perez-Riverol Y, Bandla C, Kundu DJ, Kamatchinathan S, Bai J, Hewapathirana S, John NS, Prakash A, Walzer M, Wang S, et al. The PRIDE database at 20 years: 2025 update. Nucleic Acids Res. 2025;53(D1):D543–D553. 10.1093/nar/gkae1011 PMC11701690 39494541

[105] Wickham H, François R, Henry L, Müller K, Vaughan D. dplyr: A grammar of data manipulation. 2023.

[106] Wickham H. ggplot2: Elegant graphics for data analysis. New York (NY): Springer-Verlag; 2016.

[107] Ritchie ME, Phipson B, Wu D, Hu Y, Law CW, Shi W, Smyth GK. limma powers differential expression analyses for RNA-sequencing and microarray studies. Nucleic Acids Res. 2015;43(7): Article e47. 10.1093/nar/gkv007 PMC4402510 25605792

[108] Kolde R. pheatmap: Pretty heatmaps. R package version 1.0.12. 2019.

[109] Kim HJ, Kim T, Hoffman NJ, Xiao D, James DE, Humphrey SJ, Yang P. PhosR enables processing and functional analysis of phosphoproteomic data. Cell Rep. 2021;34(8): Article 108771. 10.1016/j.celrep.2021.108771 33626354

[110] R Studio Team. RStudio: Integrated development environment for R Boston (MA): Posit Software, PBC; 2024.

[111] Wickham H, Averick M, Bryan J, Chang W, McGowan LD, François R, Grolemund G, Hayes A, Henry L, Hester J, et al. Welcome to the tidyverse. J Open Source Softw. 2019;4(43):1686.

