# Supplementary material for: A Boolean Seven-Layer Platelet Decision Model Models Consistently the Logic and Semiquantitatively the Dynamics of Platelet Activation and Inhibition
Source: Comput Struct Biotechnol J. 2026 Sep 30;35(1):0228. doi: 10.34133/csbj.0228 (PMC13624728; doi:10.34133/csbj.0228)
Supplement: Supplementary file 1 [file csbj.0228.f1.zip › SevenLayerSuppl_revised-sub-fi.docx]

# Supplementary Materials

**A Boolean Seven-Layer Platelet Decision Model integrates literature-curated, omics-data confirmed interactions to model consistently the logic and in a semiquantitative way the dynamics of platelet activation and inhibition**

Özge Osmanoglu^1,+^, Johannes Balkenhol^1,2,+^, Juan Prada^1^, Martin Kaltdorf^1^, Maral Afshar^1^, Sebastian von Mammen^3^, Katrin G. Heinze^2^, Stepan Gambaryan^4^, Harald Schulze^5^, Thomas Dandekar^1,6*.^

^1^Department of Bioinformatics, Biocenter, Julius-Maximilians-Universität Würzburg (JMU), Am Hubland, 97074 Würzburg, Germany

^2^Rudolf Virchow Center, Center for Integrative and Translational Bioimaging, Julius-Maximilians-Universität Würzburg (JMU), Josef-Schneider-Str. 2, 97080 Würzburg, Germany

^3^Department of Computer Science, Chair IX, Julius-Maximilians-Universität Würzburg (JMU), Am Hubland, 97074 Würzburg, Germany

^4^ Sechenov Institute of Evolutionary Physiology and Biochemistry of the Russian Academy of Sciences, Thorez p. 44, Saint Petersburg 194223, Russia

^5^Institute of Experimental Biomedicine, Chair I, University Hospital Würzburg, Josef-Schneider-Str. 2, 97080 Würzburg, Germany

^6^EMBL, Molecular systems biology unit, Heidelberg

^+^equal contributing first authors

*Correspondence: dandekar@biozentrum.uni-wuerzburg.de

**Contents**

**Supplementary Methods**……………………………………… …………………………2

**Supplementary Results** ..................................................................................................... 3

Supplementary Box 1: Overview of Dynamic Platelet Simulation Results ........................ 3

Suppl. Box 2: Enhancing our seven-layered platelet decision model by virtual reality

and agent-based models........................................... ……………………………………….6

Suppl. Tables (p.10): Table S1 (p. 10), Table S2 (p.12)

Suppl Figures S1-S11..p.13-22; S12/S13(age,GPVI p.23; S14/15 BSS/GTp24, log2 CC, p.25

Supplementary Discussion...................................................................................................26

Table S3 All interactions modelled (p. 31-33) References (p.33-35)

## Supplements to the Methods

*Key points on the methodological focus and its key limitations:*

1) A platelet decision model integrates available experimental data to model consistently the Boolean logic in line with all available pathophysiological experimental platelet data. The model is not quantitative, as we had insufficient kinetic data on involved protein networks. We describe only semiquantitative the dynamics of platelet activation and inhibition, i.e. the order of events and the relative strength of receptor activation or protein phosphorylation.
2) The Boolean network model (76 nodes, 115 interactions) incorporates seven layers of platelet signaling and regulatory feedback loops: (1) platelet activation stimuli resulting from vascular injury, (2) the platelet receptor layer (GPVI, GP1b-IX-V, PAR1/4, P2Y_1_/P2Y_12_, P2X1, TP, 5-HT2A, and IP) that receives signals, (3) central kinases (Src, Fyn, Lyn, PKC, PKA, PKG) that converge diverse signals, (4) the translation of signaling into changes in platelet ion homeostasis, (5) alterations in platelet shape / adhesion, (6) the activation of other platelets, and (7) the formation of a platelet plug.
3) Dynamic simulations based on available experimental data demonstrate network robustness by multiple and redundant positive feedback mechanisms. These allow rapid and irreversible aggregation and make the platelets even resilient to genetic knockouts (e.g. Trpm7). Signaling in chronic inflammatory conditions is modelled such as atherosclerosis, inflammatory arthritis and aging as well as inherited platelet disorders.
4) We explore with our Boolean network models the mechanisms behind rapid, precise platelet decisions in balancing activation and quiescence, which is essential for hemostasis. We employ Boolean models to simulate platelet behavior under various physiological and disease conditions. The model was carefully validated regarding predicting correctly the right Boolean logic network state according to available data-sets, functional studies and platelet protein interactions. This resulted in a seven-layer decision model of platelet signaling. Moreover, the detailed, well validated Boolean and semiquantitative model on platelet signaling allows to explore novel and known pharmacological intervention strategies and how they are affecting the platelet network logic.
5) However, the focus are the Boolean decision processes, we did not attempt a detailed quantitative modelling. For this there are complementing specific quantitative modelling efforts (Wangorsch et al., 2011; Breitenbach et al., 2022) including specific platelet responses (Osmanoglu et al., 2023) or SARS-infection stimulated hyperactivity or detailed response signatures or platelet phenotypes (Osmanoglu et al., 2025).

## Supplements to the Results

### Supplementary Box 1: Overview of Dynamic Platelet Simulation Results

This section reviews our computer simulations to understand how platelets behave under different conditions.

**Simulation Tool & Model:**

- We used the software Jimena and a specific model structure (platelet_network.graphml in supplementary material) to run our simulations. This helped us see how platelets make decisions and interact with each other in their signaling processes.

**Key Findings and Simulations:**

First of all, the model captures healthy state, activation and inhibition as modelled and published previously (Mischnik et al., 2014; Breitenbach et al., 2022). However, we took now into account also details of pathophysiological states and how the decision network would respond. This revealed the 7 decision layers and decision funnels helping fast reactions. Regarding pathophysiology, we looked at:

1. **Damage Response:**
   - We observed how damage to the epithelial cells (cells lining blood vessels) leads to the exposure of collagen (a protein), which then triggers platelets to activate and respond to the injury.
   - We looked at various situations:
     - Constant damage to the epithelial cells.
     - Complete absence of the epithelial cell layer.
     - Effects of chronic inflammation on platelets.
     - Impact of atherosclerosis (artery hardening due to plaque) on platelets, both with and without cell damage.
     - Influence of inflammatory arthritis on platelets, comparing situations with and without cell damage.
     - How aging affects platelets in the above conditions.
   - These situations were simulated and visualized in different figures (referred to as Figure 7, S3, S4, S5, S6, and S7).
2. **Impaired Signaling:**
   - We also studied what happens when platelets can't signal properly. For example:
     - When TRPM7 (a protein involved in magnesium and calcium signaling) is absent.
     - When platelets are isolated and don't receive external signals.
   - These conditions were also visualized in figures (Figure 1, S1, S2, S3).
3. **Genetic Differences:**
   - We simulated how platelets with different genetic makeup respond to damage or other stimuli compared to normal (wild-type) platelets. This included:
     - Platelets deficient in GPVI, a protein involved in their activation.
     - Platelets from individuals with Bernard-Soulier Syndrome, which affects how platelets stick together.
     - Platelets from individuals with Glanzmann Thrombasthenia, another condition affecting platelet function.
   - These scenarios are illustrated in figures (Figure S8, S9, S10).

**Conclusion:**

- Our model captures how platelets behave, starting from healthy state and then taking into account various factors modifying signaling responses like genetic differences, absence of certain proteins, and different disease conditions.
- We found that both the central activation process of platelets and the additional inputs (like genetic factors or disease states) are crucial to understanding how platelets respond to their environment. A decision funnel arodun the central src kinase orchestrates rapid activation, modifications include inhibitory input via cyclic nucleotides and modulation via calcium and magnesium signaling.
- Our *in silico* model mimicks the heuristic, fast and clear decision-making of platelets by including all signaling proteins and components known to have an impact according to available genetic, functional or pathophysiological data. This avoids the complexity of the tough (NP) problems in our simulations. Also, we noted that in normal (wild-type) platelets, the signaling process doesn't get stuck or halts unexpectedly (neither in experiments no in the simulations).

**Supplementary Box 2: Enhancing our seven-layered platelet decision model by virtual reality and agent-based models (ABMs):**

**Biological insights and enhancements from the ABM:** The environmental feedback loops demonstrated in the present model are conceptually central to the next step. Our simulations reveal two fundamentally distinct feedback classes that govern platelet fate: negative environmental feedback, provided by intact endothelial cells releasing PGI₂ and NO, which actively restrains activation and maintains quiescence; and positive environmental feedback, arising from granule-released ADP, serotonin, and thromboxane A₂, which amplify the activation signal and drive the system toward irreversible aggregation. Once this positive feedback crosses a critical threshold, the platelet commits to a fully activated state that can no longer be reversed by endothelial inhibitory signals alone — the biological counterpart of a latch circuit. In the single-cell SQUAD network model presented here, these environmental inputs are modelled as simplified external nodes at the receptor layer, yet this is already sufficient to reproduce the full repertoire of central platelet behaviours: quiescence, graded reversible activation, irreversible aggregation, and thrombo-inflammatory persistence. This confirms that environmental feedback is the decisive control variable, not merely a modulating factor. What a single network in isolation cannot capture, however, is what happens when thousands of such networks interact simultaneously with each other and with a spatially heterogeneous environment: the collective amplification of positive feedback across a growing platelet plug, the local depletion of inhibitory signals near the vessel wall, the mechanical feedback of shear stress on the growing thrombus, and the paracrine cross-talk between platelets, endothelial cells, and leukocytes. These emergent multi-cell dynamics require a multi-agent framework in which each platelet agent carries its own signalling network and exchanges signals through a shared, physically realistic environment — a blood vessel. Supplementary Box 2 illustrates the concrete steps already taken toward this: from platelet vessel flow through activation to platelet–endothelium interaction including thrombus formation, with spatio-temporal comparison to own intravital microscopy data. Future work will use this hierarchical ABM to study how fast inflammatory changes propagate during stroke (Wendelboe & Raskob, 2016; Raskob et al., 2014; GBD 2019 Diseases and Injuries Collaborators, 2020) before and after thrombus formation in brain tissue, and how these changes can be pharmacologically modulated (Stoll et al., 2024). This will require in vivo imaging data, such as our ongoing study on how platelet glycoprotein V spatio-temporally controls fibrin formation (Beck et al., 2023), as well as large-scale clinical data on effective interventions.

**Concrete Implementation into an ABM:** Virtual reality (VR) allows to enhance our seven-layered platelet decision model: The seven-layered signaling network is translated in its key properties into an agent-based model (ABM) where one agent represents one platelet. Using the VR we can simulate the behavior of many platelets and how they move in space and time. The VR allows hence to model and assess respective influences of the platelets, the surrounding tissue and arising pathophysiological effects. In general, a VR environment using ABM is particularly strong to elucidate spatio-temporal sequences and events. To be as realistic as possible, we integrate also image information, for example the texture of different objects, e.g. platelets, endothelial cells, vasculature (which then, in the next refinement steps influence platelet behavior). A couple of videos illustrate this:

(i) A video by Jan Grundheber using Blender on platelet vessel flow (video S1.mp4).

(ii) A video by Tobias Schmidt and Dennis Eckert using Unity on platelet activation (video S2.mkv).

(iii) A video by Johannes Balkenhol of what happens inside the platelet cell while interacting with the environment using Jimena (video S3.mp4).

For the dynamic simulations of the seven-layered decision network we use our dynamic network analysis software, Jimena. To become more realistic and allow a VR this is being translated from Java into C# to enhance its functionality in Godot and other game engines to establish a VR. This translation is a critical step in integrating sophisticated signaling networks into our virtual environment, specifically tailored to the study of platelets. By this we translate the validated signaling networks of the platelet into our virtual models looking at many platelets and their behavior in space and time. We next create machine-readable network formats for advanced analysis.

*Integration of Networks into Godot* (preferred to Unity and Blender as Godot is open source; but the procedures are similar): The networks, delivered in GraphML format, are seamlessly integrated into Godot using a specially designed C# language plugin, incorporating the Jimena Software GraphML importer. The core of our network simulation is the SQUAD method, which intuitively translates network topology into dynamic networks, altering activation patterns over time.

*Simulations for Network Control and Behavior of many platelets*: We have already developed detailed Boolean network models of central platelet signaling, inhibition and modulation. The platelet receptors and secreted platelet factors modelled, allow platelets to influence each other.

To give two central examples we currently are studying: using the Godot engine via Java and C# we turn our 7-layered model into an ABM and to reproduce the cAMP effect in the platelets in VR, and also the simple effect that the inflammatory cytokines increase in the tissue after the thrombus in the brain.

In particular, the spatio-temporal model of the ABM allows to study in a detailed way thrombus formation and packing of the platelets to and within the thrombus. cAMP concentrations are comparatively high (inhibitory for platelet activation; see Figure 2 in paper and Breitenbach et al., 2022) in the outside regions of the thrombus, but low (and hence the thrombus is more solid inside the thrombus). Critical protein interactions and concentrations, for instance the activation of the inhibitory cyclic nucleotide pathway is incorporated by Godot match and case statements.

var cAMP: int = 1

match x:

1:

\# If cAMP == 1, run this block of code

_:

\# If nothing matches, run this block of code

The activation of the inhibitory cyclic nucleotide pathway are simulated and translated into receptor activation which drive via the network logic (e.g. in Fig. 2) the behavior change such as shape changes as demonstrated in video S4. This video is no *in silico* simulation but original validation data from an experiment: A time-lapse video of intravital confocal microscopy of the superior sagittal sinus, staining of vasculature (violet, BSA-A546 and anti-CD105-A546) and platelets (green, anti-GPIV A488), showing platelets during vessel thrombosis in the brain.

The ABM allows to monitor the thrombus formation and how antithrombotic or prothrombotic factors influence this including surface effects on thrombus and endothel and effects of the endothelial cells.

Similarly, the spatio-temporal model of the ABM allows to study in a detailed way how inflammatory changes in the brain tissue happen in stroke: We can collect from the model detailed insights into cytokine effects before and after the thrombus on the brain tissue. In particular we can study the extent of the high inflammation penumbra in stroke and how this can be modulated (e.g. by drugs and T-cells) and compare tissue before and after the occlusion or inside and outside the stroke region.

With both examples, we can also show very clearly what we gain compared to our single platelet decision model (single model, fully discussed in this paper): With the spatiotemporal ABM model, we can emphasize the communication between the platelet agents as a further emergent effect, which can then be investigated in the VR model during thrombus formation, but never in our Boolean model as well as the inflammatory stimulation of the tissue by the platelets during stroke (modelled by agents representing endothelial cells).

Such simulations have been attempted before for platelets, but focused on other questions such as glycoprotein VI receptor diffusion (Tantiwong et al., 2023), circulating tumor cells (Schneider et al., 2023), or platelet–tumor interactions and metastasis progression (Uppal et al., 2014) and not on a seven-layer platelet decision model and implications for pathophysiology of platelets and disease, examined in a new agent-based, spatio-temporal model of platelets in surrounding tissue regarding thrombus formation and stroke (Nugent & Kunicki, 2017; Aurbach et al., 2019; Uppal et al., 2014; Tantiwong et al., 2023; Schneider et al., 2023). This will put us in a position to get spatio-temporal resolution and prediction on various pathologies to better understand the complex data from experiments and clinic.

## Supplementary Tables

**Table S1:** Details of simulations, perturbations for each simulation are given for each figure.

| Simulation | Perturbation | Figure | Comment |
| --- | --- | --- | --- |
| Quiescent state simulation | damage/injury: Value 0 from 0 to 1000 | Figure 3 | WT |
| Damage to the EC layer | damage/injury: Value 1 from 0 to 5 | Figure 4 | WT |
| Threshold simulation in vivo | damage/injury: Values 1 from 0 to 5 TRPM7: Value 0.5 from 0 to 1000 | Figure 5A | WT |
|  | damage/injury: Value 0.4 from 0 to 5 TRPM7: Value 0.5 from 0 to 1000 | Figure 5B | WT |
|  | damage/injury: Value 0.3 from 0 to 5 TRPM7: Value 0.5 from 0 to 1000 | Figure 5C | WT |
|  | damage/injury: Value 0.2 from 0 to 5 TRPM7: Value 0.5 from 0 to 1000 | Figure 5D | WT |
|  | damage/injury: Value 0.1 from 0 to 5 TRPM7: Value 0.5 from 0 to 1000 | Figure 5E | WT |
|  | damage/injury: Values 1 from 0 to 5 TRPM7: Value 0 from 0 to 1000 | Figure 5F | KO |
|  | damage/injury: Value 0.4 from 0 to 5 TRPM7: Value 0 from 0 to 1000 | Figure 5G | KO |
|  | damage/injury: Value 0.3 from 0 to 5 TRPM7: Value 0 from 0 to 1000 | Figure 5H | KO |
|  | damage/injury: Value 0.2 from 0 to 5 TRPM7: Value 0.5 from 0 to 1000 | Figure 5I | KO |
|  | damage/injury: Value 0.1 from 0 to 5 TRPM7: Value 0 from 0 to 1000 | Figure 5J | KO |
| Threshold simulation in vitro | damage/injury: Values 1 from 0 to 5 TRPM7: Value 0.5 from 0 to 1000 | Figure S2A | WT |
|  | damage/injury: Value 0.4 from 0 to 5 TRPM7: Value 0.5 from 0 to 1000 | Figure S2B | WT |
|  | damage/injury: Value 0.31 from 0 to 5 TRPM7: Value 0.5 from 0 to 1000 | Figure S2C | WT |
|  | damage/injury: Value 0.2 from 0 to 5 TRPM7: Value 0.5 from 0 to 1000 | Figure S2D | WT |
|  | damage/injury: Value 0.11 from 0 to 5 TRPM7: Value 0.5 from 0 to 1000 | Figure S2E | WT |
|  | damage/injury: Values 1 from 0 to 5 TRPM7: Value 0 from 0 to 1000 | Figure S2F | KO |
|  | damage/injury: Value 0.4 from 0 to 5 TRPM7: Value 0 from 0 to 1000 | Figure S2G | KO |
|  | damage/injury: Value 0.31 from 0 to 5 TRPM7: Value 0 from 0 to 1000 | Figure S2H | KO |
|  | damage/injury: Value 0.2 from 0 to 5 TRPM7: Value 0.5 from 0 to 1000 | Figure S2I | KO |
|  | damage/injury: Value 0.11 from 0 to 5 TRPM7: Value 0 from 0 to 1000 | Figure S2J | KO |
| Atherosclerosis | kinase: Value 0.5 from 0 to 1000 atherosclerosis: Value 0 from 0 to 1000 | Figure 7A | WT |
|  | kinase: Value 0.5 from 0 to 1000 atherosclerosis: Value 0 from 0 to 1000 damage/injury: Value 1 from 0 to 5 | Figure 7B | WT |
|  | kinase: Value 0.5 from 0 to 1000 atherosclerosis: Value 0.4 from 0 to 1000 | Figure 7C | WT |
|  | kinase: Value 0.5 from 0 to 1000 atherosclerosis: Value 0.4 from 0 to 1000 damage/injury: Value 1 from 0 to 5 | Figure 7D | WT |
|  | kinase: Value 0.5 from 0 to 1000 atherosclerosis: Value 0.52 from 0 to 1000 | Figure 7E | WT |
|  | kinase: Value 0.5 from 0 to 1000 atherosclerosis: Value 0.52 from 0 to 1000 damage/injury: Value 1 from 0 to 5 | Figure 7F | WT |
|  | kinase: Value 0.5 from 0 to 1000 atherosclerosis: Value 0.7 from 0 to 1000 | Figure 7G | WT |
|  | kinase: Value 0.5 from 0 to 1000 atherosclerosis: Value 0.7 from 0 to 1000 damage/injury: Value 1 from 0 to 5 | Figure 7H | WT |
| Inflammatory arthritis (IA) | kinase: Value 0.5 from 0 to 1000 IA: Value 0.5 from 0 to 1000 | Figure S6A | WT |
|  | kinase: Value 0.5 from 0 to 1000 IA: Value 0.5 from 0 to 1000 damage/injury: Value 1 from 0 to 5 | Figure S6B | WT |
|  | kinase: Value 0.5 from 0 to 1000 IA: Value 0.52 from 0 to 1000 | Figure S6C | WT |
|  | kinase: Value 0.5 from 0 to 1000 IA: Value 0.52 from 0 to 1000 damage/injury: Value 1 from 0 to 5 | Figure S6D | WT |
|  | kinase: Value 0.5 from 0 to 1000 IA: Value 0.58 from 0 to 1000 | Figure S6E | WT |
|  | kinase: Value 0.5 from 0 to 1000 IA: Value 0.58 from 0 to 1000 damage/injury: Value 1 from 0 to 5 | Figure S6F | WT |
| Aging | kinase: Value 0.5 from 0 to 1000 aging: Value 0.5 from 0 to 1000 | Figure S7A | WT |
|  | kinase: Value 0.5 from 0 to 1000 aging: Value 0.5 from 0 to 1000 damage/injury: Value 1 from 0 to 5 | Figure S7B | WT |
|  | kinase: Value 0.5 from 0 to 1000 aging: Value 0.52 from 0 to 1000 | Figure S7C | WT |
|  | kinase: Value 0.5 from 0 to 1000 aging: Value 0.52 from 0 to 1000 damage/injury: Value 1 from 0 to 5 | Figure S7D | WT |
|  | kinase: Value 0.5 from 0 to 1000 aging: Value 0.58 from 0 to 1000 | Figure S7E | WT |
|  | kinase: Value 0.5 from 0 to 1000 aging: Value 0.58 from 0 to 1000 damage/injury: Value 1 from 0 to 5 | Figure S7F | WT |
| GPVI-deficiency | kinase: Value 0.5 from 0 to 1000 damage/injury: Value 1 from 0 to 5 | Figure S8A | WT |
|  | kinase: Value 0.5 from 0 to 1000 damage/injury: Value 1 from 0 to 5 GPVI: Value 0 from 0 to 1000 | Figure S8B | GPVI-deficiency |
| Bernard-Soulier Syndrome (BSS) | kinase: Value 0.5 from 0 to 1000  vWF: Value 1 from 0 to 5 | Figure S9A | WT - vWF stimulation |
|  | kinase: Value 0.5 from 0 to 1000 vWF: Value 1 from 0 to 5 GP1b-IX-V: Value 0 from 0 to 1000 | Figure S9B | BSS - vWF stimulation |
|  | kinase: Value 0.5 from 0 to 1000  collagen: Value 1 from 0 to 5 | Figure S9C | WT - collagen stimulation |
|  | kinase: Value 0.5 from 0 to 1000 collagen: Value 1 from 0 to 5 GP1b-IX-V: Value 0 from 0 to 1000 | Figure S9D | BSS - collagen stimulation |
|  | kinase: Value 0.5 from 0 to 1000  ADP: Value 1 from 0 to 5 | Figure S9E | WT - ADP stimulation |
|  | kinase: Value 0.5 from 0 to 1000 ADP: Value 1 from 0 to 5 GP1b-IX-V: Value 0 from 0 to 1000 | Figure S9F | BSS - ADP stimulation |
| Glanzmann Thrombasthenia (GT) | kinase: Value 0.5 from 0 to 1000 damage/injury: Value 1 from 0 to 5 | Figure S10A | WT |
|  | kinase: Value 0.5 from 0 to 1000 damage/injury: Value 1 from 0 to 5 αIIbβ3: Value 0 from 0 to 1000 | Figure S10B | GT |

**Table S2**: Decision cascades in the platelet: Efficient and robust but failing with aging

The platelet cascade is shown in Figure 2. Three features are combined here and support our considerations of decision processes as fast, robust heuristics versus exact mathematical decisions which may, however, sometimes take unforeseeably long to execute:

**I) Efficient platelet signaling cascade:** The platelet signaling cascade is fast and executes decisions very reliably according to all available evidence. Even multiple protein knockouts do not affect the central signaling process---it stays robust and fast. Concrete data that there is a central signaling backbone for efficient signaling transmission and that unless the central backbone is hit, otherwise processing is not harmed, both in mouse and man highly similar and well conserved signaling cascade and also for inhibitory signals (Balkenhol et al., 2020). In supplement, we show that TRPM7 KO affects signaling in model1 (without feedbacks and injury and healthy EC layer additions), but when feedbacks are involved, it does not change much because the signaling is more robust.

**II) Robust decision processes, decision funnels and activation control:** Platelet decision heuristics involve the SRC kinase and a bistability switch to ensure robust, fast, and direct decisions. This was confirmed by a combination of light-scattering-based thrombocyte aggregation data, western blot and calcium measurements (Mischnik et al., 2014). Evidence 2: Dynamic control centrality describes relaying functions as observed in signaling cascades of the SRC kinase (Karl and Dandekar, 2015b). The activating cascade is well conserved and robust, input from neighbor proteins modulates the signal, while the cytoskeleton executes the platelet activation as a subnetwork (Balkenhol *et al.*, 2020).

**III) However, shift in balance from fast, reliable execution by constant stress leads to thrombo-inflammation**

High stress, many activating inputs and inhibition signals at the same time lead to chronic inflammation, and too many cellular accidents due to aging result in chronic platelet pre-activation and thrombo-inflammation. This is almost a necessary consequence of the optimized cascade for blood-clotting in combination with infection protection, but with aging this leads to chronic inflammation: The rapid and robust decision due to positive feedbacks, parallel signaling pathways etc. works only well because it's controlled by self-brakes and EC control. When there is continuous activation from outside, although there is no injury, like in cancer and chronic inflammation (psoriasis, atherosclerosis, lupus etc.) and thrombo-inflammation (COVID-19, bacterial infections etc.), this leads to overactivation and trigger pathologies like thromboembolism etc. This effect is shown *in silico* in a simulation in the supplement.

## Supplementary Figures

**Figure S1: Platelet signaling model without external control (representing *in vitro* conditions).** Topology of the underlying signaling network for simulations of in vitro experiments. The essential elements of the network are displayed in nodes, interconnected with either activating (green) or inhibiting (red) arrows. The difference from the model shown in the manuscript is the absence of amplification loops and internal and external inhibitory control.

**A**

**B**

**C**

**Figure S2: Comparison of gene expression changes of the platelet CC nodes in (A) non-ICU (B) ICU and (C) NSCLC**. red: upregulated (log2FC > 0.58), pink: slightly upregulated (log2FC > 0.3), blue: downregulated (log2FC < -0.58), and light blue: slightly downregulated (log2FC < -0.3), gray: not significant. Yellow framing indicates subtle but significant regulation (-0.3 < log2FC < 0.3). For protein complexes or different isoforms, only significant expression changes are visualized (e.g., for GPIb-IX-V and PI3K in panel A).

**Figure S3: Threshold simulation without external control (representing in vitro conditions), panels A–J.** TRPM7 Kinase domain is set as constitutively active in wild-type (WT, left) and 0 in TRPM7 knockout (-/-, right). Activation of platelet cascade by collagen is simulated for two conditions of TRPM7: wild type (WT) in which TRPM7 is set to medium strength of 0.5 due to unknown kinetics of the kinase, and knockout (TRPM7^-/-^) in which the kinase is set to 0. In both cases, the initial activation of PLC pathway leads to an increase in Ca2+ levels, and followingly, the ion channels for magnesium and calcium (e.g., TRPM7) are activated, balancing the Ca2+ levels with Mg2+, and the network then reaches the state of irreversible aggregation. When TRPM7 is inactivated (TRPM7^-/-^), there is a slight delay in the activation of the cascade, and in the subsequent Ca2+ increase, the network stays active for a shorter duration.

**Figure S4: Constant damage simulation.** Platelet activation persists, although some of the intrinsic self-inhibition mechanisms (e.g., RGS18-cGMP signaling) respond to platelet activation by trying to suppress it. However, continuous external input leads to repeated platelet activation (damage/injury: Value 1 from 0 to 1000).

**Figure S5: EC absence simulation.** Platelet activation persists similarly to constant damage simulation, although some of the intrinsic self-inhibition mechanisms (e.g., RGS18-cGMP signaling) respond to platelet activation by trying to suppress it. However, lack of EC control leads to repeated platelet activation, even in the absence of damage (Healthy EC: Value 0 from 0 to 1000).

**Figure S6: Chronic inflammation diseases integrated in the model.** Effect of inflammation on platelet signaling is shown with a focus on atherosclerosis, inflammatory arthritis (IA), and aging. In atherosclerosis, endothelial cells (ECs) secrete vWF and promote platelet adhesion. In return, activated platelets release CD40L, which induces ECs to take on a more pro-inflammatory and pro-thrombotic profile, leading to more platelet activation, e.g., via inhibition of platelet-inhibitory cGMP signaling. In IA, ECs also show a pro-inflammatory and pro-thrombotic profile, and this increases platelet activation mainly via ADP signaling. Factors like CD40L inhibit the ecto-ADPase of ECs, promoting the ADP signaling even more. Lastly, in aging, although metabolic reprogramming is a main way of regulating platelets, we here only show the effects on signaling. This is exerted mainly via TNF-α secretion by monocytes and also via increased expression of platelet adhesion receptors, like GP1b-IX-V.

control.

**A**

**B**

**F**

**G**

**H**

**I**

**J**

**C**

**D**

**E**

**Figure S7: Threshold analysis of TRPM7 knockout without external control. TRPM7-/-) threshold analysis and simulation without external control layers.** Stimulation of the platelet signaling network with different levels of collagen input. TRPM7 Kinase domain is set as constitutively active in wild-type (WT, upper row), and 0 in TRPM7 knockout (-/-, lower row). Different activity levels for platelet responses (see colour legend below subpanels) and Trpm7 receptor (violet) are traced. Threshold analysis determined the intermediate value of collagen to be 0.2 for WT and 0.4 for TRPM7-/- (defined as the minimum collagen stimulus required to reach a full irreversible aggregation (value 1)) and the lower value to be 0.11 for WT and 0.31 for TRPM7-/- (the minimum collagen stimulus to activate the irreversible aggregation to a higher value than the collagen input).

**A**

**B**

**C**

**D**

**E**

**F**

**Figure S8: (A–F) Simulation of Bernard-Soulier Syndrome (BSS)** (right) compared to wild-type simulations (left) in response to different agonists. Platelet aggregation in response to VWF in (A) wild-type platelets is lost in (B) BSS. However, other agonist-induced platelet aggregation responses are not affected, including (C-D) collagen, (E-F) ADP.

**A**

**B**

**C**

**D**

**E**

**G**

**F**

**H**

**Figure S9: Atherosclerosis simulation with damage input (right) and without damage (left).** Values below 0.52 for atherosclerosis do not lead to any aberrations in platelet aggregation. Values between 0.52 and 0.7 only lead to aberrant aggregation when there is a damage input. Values equal to and greater than 0.7 lead to aberrant aggregation even though no damage is present.

**A**

**B**

**C**

**D**

**E**

**F**

**Figure S10: Aging simulation with damage input (right) and without damage (left).** Values below 0.52 for aging do not lead to any aberrations in platelet aggregation. Values between 0.52 and 0.58 only lead to aberrant aggregation when there is a damaged input. Values equal to or bigger than 0.58 lead to aberrant aggregation even though no damage is present.

**Figure S11: Inflammatory arthritis (IA) simulation with damage input (right) and without damage (left).** Values below 0.52 for IA do not lead to any aberrations in platelet aggregation. Values between 0.52 and 0.58 only lead to aberrant aggregation when there is a damaged input. Values equal to or bigger than 0.58 lead to aberrant aggregation even though no damage is present.

**Figure S12: Aging simulation with damage input (right) and without damage (left).** Values below 0.52 for aging do not lead to any aberrations in platelet aggregation. Values between 0.52 and 0.58 only lead to aberrant aggregation when there is a damaged input. Values equal to or bigger than 0.58 lead to aberrant aggregation even though no damage is present.

**Figure S13: Simulation of GPVI-deficiency (right) compared to wild-type simulations (left) in response to damage.** Platelet aggregation in response to collagen in wild-type platelets (A) is lost in GPVI deficiency (B).

**Figure S14: Simulation of Bernard-Soulier Syndrome (BSS) (right) compared to wild-type simulations (left) in response to different agonists.** Platelet aggregation in response to vWF in wild-type platelets (A) is lost in BSS (B). However, other agonist-induced platelet aggregation responses are not affected (collagen (C-D), ADP (E-F)) in BSS.

**Figure S15: Simulation of Glanzmann Thrombasthenia (GT) (right) compared to wild-type simulations (left) in response to damage.** GT is caused by loss-of-function mutations in αIIbβ3, leading to absent or severely reduced platelet aggregation with all agonists. Consistent with this, platelet aggregation in response to damage in wild-type platelets (A) is absent in GT (B), reflecting the loss of αIIbβ3-mediated outside-in signaling regardless of stimulus type.

**Figure S16: Cross-dataset log2 fold-change comparison of platelet CC nodes across disease conditions.** Log2 fold-changes (log2FC) of platelet central cascade (CC) node genes are shown across 17 disease conditions from published datasets, grouped by disease category: cancer (Breast, CRC, Pancreas, NSCLC, GBM, Hepatocellular carcinoma, Immature platelets), cardiovascular (STEMI, NSTEMI), infection (non-viral Sepsis, Sepsis, COVID-19 non-ICU, COVID-19 ICU, COVID-19 Dataset 3), and reticulated platelets. Positive values (warm colors) indicate upregulation; negative values (cool colors) indicate downregulation relative to healthy controls. The heatmap enables cross-condition comparison of which CC nodes are consistently dysregulated across disease states. Data sources: Osmanoglu et al. (2023) for COVID-19 and Cancer datasets; additional datasets from the literature as detailed in Table S2.

## Supplementary Discussion

**Simulated network decision dynamics of platelet signalling agrees with physiology.**

When there is an injury, this state is rapidly transformed into platelet activation (**Fig. 4**). As a result of the injury, subendothelial collagen is exposed, and platelet inhibitory signaling from the healthy EC layer is interrupted until the damage is repaired. Here, initial activation arises from the binding of the collagen to the platelet receptors at the injury site (Stalker et al., 2012). This initial adhesion to collagen fibers is carried by the integrin α2β1 and the glycoprotein GPVI (ITAM signaling) (only GPVI is represented in **Fig. 4**) (Yeung et al., 2018). At high shear, binding of the GPIb-IX-V complex to vWF is also needed for stable adhesion (Estevez and Du, 2017). Adhesion initiates downstream signaling via PLCγ (denoted as PLC) and PI3Kβ (PI3K), leading to platelet activation (Muehlenkamp and Brausch, 2019; Yeung *et al.*, 2018). Platelet activation is followed by shape change, granule release (e.g., ADP from dense granules), and thromboxane2 (TXA2) generation via arachidonic acid (AA) cascade (Linkous and Yazlovitskaya, 2010). Furthermore, upon adhesion, thrombin is formed along the coagulation pathway via prothrombin (F2) on the surface of activated platelets (Yeung *et al.*, 2018).

Activation of PLC promotes an increase in the cytosolic calcium (Ca2+), activating Rap1B and RAC1, leading to cytoskeletal rearrangements involved in shape change. The platelets change from their natural discoid shape to a more extended shape via the formation of lamellipodia and filopodia. Similarly, Rac1 activation activates α-granule secretion (Golebiewska and Poole, 2013). Within α-granules, proplatelet agents like ADP, ATP, and serotonin (5-HT) are also released.

Granule release and TxA2 production leads to the amplification of the platelet activation signal by activating GPCRs (PAR1/4, P2Y1, P2Y12, P2X1, TP, 5-HT2A) and their downstream signaling through various G proteins. Thrombin receptor PAR1/4, ADP receptor P2Y1, TXA2 receptor TP, and serotonin receptor 5-HT2A act through Gα_q_, which activates PLCβ. Gβγ is associated with receptors PAR1/4 and P2Y12 and activates PI3Kγ, while Gα_12/13_ coupled to PAR1/4 and TP activates ARHGEF1 (followingly RHO-A). Moreover, Gα_i_ coupled to P2Y12 inhibits AC and thus lifts platelet inhibition exerted by the cAMP signaling pathway (Li et al., 2010; Lopez-Vilchez et al., 2009; Nagy and Smolenski, 2018; Oury et al., 2015; Yeung *et al.*, 2018).

These signal transduction and amplification events during platelet activation then convert αIIbβ3 (a2b3 in the network) to its high-affinity active state for binding extracellular ligands such as fibrinogen or vWF. The αIIbβ3-fibrinogen or αIIbβ3-vWF complexes act as bridges between adjacent activated platelets and thus lead to platelet aggregation.

The positive feedback via granule release ensures a fast platelet aggregation that is needed in a time of injury. This robust mechanism (robustness against TRPM7 KO shown in **Fig. 5-6**) of rapidly recruiting more and more platelets ensures a fast response by amplifying the initial signal (Li *et al.*, 2010).

On the other hand, platelets do not aggregate without an end. The activation is terminated after the damage is repaired. This is achieved by various mechanisms, exemplified in our model by the negative feedback occurring in and outside the platelet. As the internal self-brake mechanism, we show RGS18, which is normally activated by platelet inhibitors (like cyclic nucleotides) and inhibits G protein signaling (Makhoul et al., 2018). In the network, we represent the internal mechanism by the inhibition that acts directly on PLC as a direct downstream of G protein signaling. The external self-brake also acts through cyclic nucleotides and functions through the mechanosensitive cGMP signaling. This mechanism includes the inhibition of thrombus growth by activation of guanylyl cyclase mechanically by the increasing shear stress in the bloodstream with the size of the clot formed (Feil et al., 2022).

#### **Decision funnels in platelets: effects of constant stimulation and chronic inflammation.**

The feedback loop via the external environment allows platelets to stabilize decision funnels or preferred decisions. The mechanisms for rapid decision indeed allow platelets to act rapidly, smoothly, and in a P-problem-like fashion: there is no stalling, and there is not much variation in decision time (in platelets measured by aggregometry). Hence, the platelets make a robust cellular decision. As a result, per time unit or biological system, there happens a surprisingly low amount of error. This also is expected to decrease the possible effect of a single mistake or mutation in a protein on the signaling outcome, which keeps hemostasis, an essential mechanism for survival, unaffected and functional. These single mistakes can have a more profound effect in the absence of signal amplification or the absence of the biological microenvironment (**Fig. 6 and S1-2**). On the other hand, under continuous stress, these robust and fast platelet decisions may lead to thrombo-inflammation because of constantly activating stimuli such as in diabetes, inflammation, infection, aging, or other conditions that increase oxidative stress and trigger various pathologies (**Fig. 7 and S5-7**, **Table S2**).

In thrombo-inflammation, the lack of EC control on thrombosis and inflammation leads to hyperactivation of platelets. As mentioned before, endothelial cells have an anti-thrombotic control over platelet function via various mechanisms. Likewise, ECs have an anti-inflammatory profile under healthy conditions. During thrombo-inflammation, inflammatory processes can shift the balance between pro- and anticoagulant properties of endothelium. This control can be lost, leading to continuous activation of platelets and recruitment of immune cells to the endothelium. Since platelets respond strongly to environmental stimuli, once the EC control is absent, they are hyperactive, and the environmental stimuli lead to their (self)-activating interaction with the endothelium and with various immune cells (Klavina et al., 2022; Sharma et al., 2022). Our simulations show recurring platelet activation in a loop in the continuous presence of procoagulant stimuli or the absence of EC control (**Fig. S3-4**).

- The role of platelets in chronic inflammation differs from the mechanisms in acute inflammation summarized above. In early phases of acute inflammation in response to bacterial or viral infection, platelet activation and coagulation help contain the pathogen dissemination at the site, partly by activating neutrophil NETosis (Stark and Massberg, 2021). In later stages, platelet activation inhibits neutrophils and suppresses TNF-α secretion from monocytes (Corken et al., 2014), leading to the resolution of the inflammation. The same receptor, GP1b, sustains both pro- and anti-inflammatory characteristics of platelets.

In chronic inflammation, the decision funnel switches in platelets from thrombosis and aggregation to favoring inflammation and thrombo-inflammation.

Hence, a change in platelets from pro-inflammatory to anti-inflammatory character is absent under these conditions (Corken *et al.*, 2014) as well as in ECs. The inflammation is associated with an increased risk of thrombosis (Stark and Massberg, 2021). More detailed literature comparison in the suppemental discussion.

#### **Shifting decision funnels and platelet system state in disease**

In atherosclerosis, which is a chronic inflammation disease, interaction of platelets with endothelial cells and immune cells promotes the atherosclerosis (Chen et al., 2020). Platelet adherence to the endothelium occurs through GP1bα and αIIbβ3 initiating the plaque formation (Stark and Massberg, 2021). Platelets further interact with the plaque via their GPVI receptors, earlier with laminin, and with collagen once the plaque raptures. GPVI signaling is essential in atherothrombosis and its inhibition was shown to inhibit thrombus formation (Jamasbi et al., 2015). Platelets do not contribute to the chronic inflammation in atherosclerosis only by their thrombotic function. They also recruit neutrophils and promote the adhesion of monocytes to the atherosclerotic endothelium and release cytokines and chemokines, increasing inflammation (such as CD154, IL1β, CCL5 (Klavina *et al.*, 2022), CXCL4, CXCL5, MIF (Stark and Massberg, 2021)). Platelets interaction with leukocytes and endothelial cells is mediated majorly by the platelet P-selectin (Klavina *et al.*, 2022) e.g., binding to PSGL1 on neutrophils, or interactions between platelet HMGB1 and neutrophil RAGE, platelet GP1bα and neutrophil integrin aMβ2 (MAC1) promoting neutrophil recruitment and NETosis. In return, neutrophils further activate platelets by the antimicrobial cathelicidin LL37/CRAMP they secrete via platelet GPVI signaling (Stark and Massberg, 2021).

What leads to artherothrombosis (plaque rupture) is continuous low-grade inflammation, during which ECs secrete vWF which promotes platelets' adhesion by their Gp1b receptor (GPIb-IV-X complex) activating platelet signaling. Platelet activation, in return, ends in release of factors like CD40 ligand, which induces ECs to produce more ROS, adhesion and inflammatory molecules such as IL1β, which induces ECs to express more adhesion proteins and promotes the adhesion of neutrophils and monocytes to the endothelium. At the same time, activated platelets also release PF-4 and more ROS. Production of ROS is observed to be driven via upregulating of NOX and COX-1, which then scavenges the NO in the environment, which normally is taken up by platelets and mediates inhibitory platelet signaling via cGMP. When the NO is scavenged by ROS, the absence of inhibitory signaling leads to more platelet activation and aggregation (Davi and Patrono, 2007). We modeled the effect of Ecs during the low-grade inflammation in atherosclerosis by adding the mechanisms summarized above (**Fig. 7**). We simulated different levels of atherosclerosis both in the absence of a damage/injury and when there is a damage stimulus. In all simulations, we started with a fully active "healthy EC layer" to simulate the changing balance between the healthy and inflamed EC layer. We introduced a damage stimulus at timepoint 10 to see the effects on a situation, where both inflammation and EC control is present at different levels. In the absence of damage, our simulations showed atherothrombosis only at high levels of inflammation caused by atherosclerosis (atherosclerosis ≥ 0.7). Medium levels of inflammation (0.52≤atherosclerosis<0.7) led to aberrant platelet aggregation only in presence of a damage stimulus. In other words, in the medium range of atherosclerosis, a damage stimulus was enough to shift the balance from healthy EC to inflamed EC. However, low-grade inflammation (atherosclerosis < 0.52) did not result in atherothrombosis even in presence of a damage stimulus, which failed to disrupt a healthy EC layer and its anti-thrombotic profile (**Fig. 7**).

Another chronic inflammatory disease, in which platelets show increased activity is inflammatory arthritis (IA). This activity is attributed to ADP signaling. ADP normally activates platelets by binding to P2Y12, which is a G-protein-coupled receptor. Downstream signaling leads to platelet activation and release of factors including CD40-ligand (CD40L), which is also induced by increased TNF-α levels in IA. CD40L, in return, induces Ecs to produce and secrete more pro-inflammatory factors and leads to the activation of more platelets by inhibiting the ecto-ADPase of endothelial cells resulting in higher amounts of available ADP in the bloodstream (Mac Mullan et al., 2010). We integrated the interplay between the IA-induced pro-thrombotic EC profile via ADP signaling and the platelet hyperactivation into our model (Fig. SÖ8) and simulated the effects of different levels of IA on platelet aggregation. We observed a similar trend as in atherosclerosis; however, IA exerted a stronger effect on platelet aggregation even at lower/medium levels (IA≥0.58) and ended up in full activation of platelet aggregation even in the absence of a damage input (**Fig. S6**).

High levels of TNF-α are also observed in the chronic inflammation during aging and is secreted by monocytes (Gu and Dayal, 2020). In return, activated platelets promote production of more cytokines by monocytes and also express surface proteins that increase the reactivity of platelets (Price et al., 2020). These platelets also showed higher surface expression of Gp1bα and GPIX compared to platelets from young mice (Gu and Dayal, 2020); they have in Fig. 6 a heatmap for scRNAseq data for platelet activation genes in old and young platelets). High levels of TNF-α asserts its effects by metabolic programming of the platelets, which leads to changes in pathways involved in mitochondrial processes. These effects of TNF-α were shown to be on thrombopoiesis and megakaryopoiesis rather than platelet signaling directly (Davizon-Castillo et al., 2019). We represented the effect of aging on expression of platelet receptors Gp1bα and GPIX and inflammation via TNF-α levels at the signaling level (metabolic reprogramming is not represented in our model) and our simulations showed almost identical results as platelet hyperactivation caused by IA-induced inflammation (**Fig. S7**).

#### **Decision funnel:** Platelet decisions happen in decision funnels, which helps model and understand platelet pathophysiology. **Hyperactivation**

Heuristic decision processes keep the platelets' response and cellular decision robust and fast by cascading activation of platelet signaling in the presence of external signals. Decision processes are not perfect, and decision funnels may shift under long-term stress, leading to disbalance in the platelet. Here, hyperfunctioning of platelets is more common than hypo-functioning, and particularly, the state of thrombo-inflammation is often reached.

Inherited platelet disorders easily trip the platelet signaling balance, majorly concerning the size of the platelets and their function. The first type of platelet disorder can lead to low counts of platelets (thrombocytopenia) or high counts of platelets (thrombocytosis) and is mostly caused by mutations in genes associated with megakaryopoiesis and thrombopoiesis. On the other hand, diseases concerning the platelet function are associated with hypofunctional platelets due to mutations in major platelet receptors like GPVI (**phenotype**: absent or severely reduced platelet aggregation in response to collagen and other GPVI agonists, confirmed/modeled by our simulations in **Fig. S8**), GP1b-GPIX-GPV (Bernard-Soulier Syndrome, **phenotype**: absence of ristocetin-induced platelet aggregation, reduced response to other agonists like collagen and ADP (Berndt and Andrews, 2011), reproduced by our simulations in **Fig. S9**), and αIIbβ3(Glanzmann Thrombasthenia (GT), phenotype: absent or severely reduced LTA with all agonists (ADP, TxA2, collagen, thrombin), confirmed by our simulations in **Fig. S10**) or in genes associated with platelet granules and signaling (Palma-Barqueros et al., 2021). However, inherited platelet disorders spanning 60 diseases are rare and vary from person to person in the clinical outcome (Bastida et al., 2019). A recent study showed a 3:10^4^ prevalence for clinically relevant loss-of-function variants in platelet genes (Oved et al., 2021). On the other hand, thrombosis, caused by hyperfunctioning of the platelets, has been described as a major cause of death in the world, accounting for 25% of the deaths worldwide in 2010 (Wendelboe and Raskob, 2016). Thrombosis is the top cause of cardiovascular disorders like ischemic heart disease, stroke, and venous thromboembolism (VTE) (Raskob et al., 2014). Among the 42.0 million deaths from non-communicable diseases in 2019, ischemic heart disease caused 9.14 million and stroke caused 3.29 million deaths accounting for 29.5% of the deaths worldwide (Diseases and Injuries, 2020). Additionally, VTE was shown to have a yearly incidence rate of 1-2:1000 in Europe and the USA (Lutsey and Zakai, 2023). All combined account for a higher incidence rate than hypo-functioning platelet disorders, and there are far higher numbers of thrombotic events in addition to the deaths caused by the diseases listed, they are not sufficiently treated and not included in the incidence rates. We can confidently say that the hyperfunctioning of platelets is more common than hypo-functioning and this also is explained by how the platelet signaling is controlled to focusing on fast and cascading activation of platelet signaling in presence of external stimuli. This can also explain the weak effect of specific gene KOs on our model (except the receptors (**Fig. S8-10**)) in the presence of an external stimulus, e.g., damage to the endothelium. On the other hand, if we have a continuous stimulus or overactivation of a protein, the platelet activation status is irreversibly sustained at its maximum. This is what happens in pathologies involving hyperactivation of platelets, in which the sustained external stimulus or inhibited external EC control leads to aberrant activation of platelets. The second half of the cycle is closed by platelets activating the cause of their activation, be it inflammation or cancer.

**Table S3:** References for the interactions in the platelet model. To save space, the references are concisely given as Pubmed IDs (PMID).

| node1 | int | node2 | ref |
| --- | --- | --- | --- |
| TRPM7 | + | Mg2+ | 29146750 |
| TP | + | ARHGEF1 | 14528298, 17298951 |
| TP | + | PLC | 29925522 |
| PAR1/4 | + | PLC | 29925522 |
| GPVI | + | PLC | 29925522 |
| P2Y1 | + | PLC | 29925522 |
| PLC | + | IP3 | 29925522, 29146750 |
| PLC | + | DAG | 29925522 |
| PI3K | + | AKT | 29925522, 30046761 |
| SRC | + | AKT | 23463387, 20586915 |
| DAG | + | CALDAG | 29925522 |
| PKC | + | SRC | 15314167 |
| PKC | + | RAP1 | 16979556 |
| RAP1 | + | TALIN1 | 26929147, 35721481 |
| RHO-A | + | ROCK1 | 14660612, 23121917 |
| P2Y12 | + | PI3K | 29925522 |
| IP | + | AC | 29925522, 30046761 |
| AC | + | cAMP | 29925522, 30046761 |
| cAMP | + | PKA | 28228483, 30046761 |
| PKA | + | VASP | 16197368 |
| P2X1 | + | Ca2+ | 25709760 |
| Mg2+ | - | Ca2+ | 29146750 |
| PKA | - | SRC | 23463387, 12606547 |
| PKA | - | RAP1 | 26929147 |
| P2Y12 | - | AC | 29925522 |
| PAR1/4 | + | ARHGEF1 | 29925522, 17298951 |
| PKA | - | IP3R | 26929147, 30046761 |
| PKA | - | RHO-A | 24100445, 30046761 |
| ATP | + | P2X1 | 25709760 |
| TXA2 | + | TP | 29925522 |
| THROMBIN | + | PAR1/4 | 29925522 |
| COLLAGEN | + | GPVI | 29925522 |
| ADP | + | P2Y1 | 29925522 |
| ADP | + | P2Y12 | 29925522 |
| PGI2 | + | IP | 29925522 |
| Ca2+ | + | CALDAG | 29925522 |
| PAR1/4 | + | PI3K | 29925522 |
| 5-HT | + | 5-HT2A | 19541671, 21071698 |
| 5-HT2A | + | PLC | 21071698 |
| KINASE | + | PLC | 29146750, 22759789 |
| Ca2+ | + | STIM1 | 29146750, 16582901, 18559454 |
| DAG | + | TRPC6 | 9930701 |
| TRPC6 | + | Ca2+ | 9930701 |
| ORAI1 | + | Ca2+ | 29146750 |
| PKC | + | Other Mg2+ Transporters | 23727583, 15804357 |
| PKC | + | TRPM7 | there is evidence for PKA --> TRPM7 in 15069188 |
| PKC | - | TRPC6 | 20961851, 16772730, 11278449, 12721302 |
| a2b3 | + | Aggregation | 29550521, 15696195 |
| RAP1 | + | RAC1 | 24588354 |
| Other Mg2+ Transporters | + | Mg2+ | 15804357 |
| Granule release | + | ADPr | 29925522, 24588354 |
| Granule release | + | ATPr | 29925522, 24588354 |
| Granule release | + | 5-HTr | 29925522, 24588354 |
| VASP | - | Shape Change | 16197368, 26929147 |
| MAPKC | + | PLA2 | 20642808, 23620575 |
| PLA2 | + | TXA2r | 20642808 |
| Mg2+ | - | TRPM7 | 29500477, 17217066, 11385574, 22759789 |
| Other Mg2+ Transporters | + | Ca2+ |  |
| IP3 | + | IP3R | 29925522, 28228483, 29146750 |
| IP3R | + | Ca2+ | 29925522, 28228483, 29146750 |
| sGC | + | cGMP | 20929147, 30046761 |
| cGMP | + | PKG | 20929147 |
| PKG | - | RAP1 | 20929147 |
| PKG | - | IP3R | 20929147 |
| PI3K | + | PLC | hsa04611 |
| GPVI | + | PI3K | 30429532 |
| AKT | + | MAPKC | 29925522, 28228483 |
| DAG | + | PKC | 29925522 |
| CALDAG | + | RAP1 | 29925522, 26929147 |
| NO | + | sGC | 26929147, 30046761 |
| STIM1 | + | ORAI1 | 29146750, 16582901, 18559454 |
| ARHGEF1 | + | RHO-A | 14528298, 17298951 |
| TALIN1 | + | a2b3 | 26929147, 35721481 |
| RAC1 | + | Granule release | 24588354 |
| ADPr | + | ADP | released |
| ATPr | + | ATP | released |
| TXA2r | + | TXA2 | released |
| 5-HTr |  | 5-HT | released |
| PKG | - | RHO-A | 29550521 |
| Aggregation | + | Thrombus growth | 30327468 |
| Thrombus growth | + | shear stress | 30327468 |
| shear stress | + | sGC | 30327468 |
| PKA |  | RGS18 | 30046761 |
| PKG |  | RGS18 | 30046761 |
| RGS18 | - | PLC | 30046761 |
| healthy EC layer | - | THROMBIN | 26481314, 30429532 |
| healthy EC layer | + | PGI2 | 26481314 |
| healthy EC layer | + | NO | 26481314 |
| damage/injury | - | healthy EC layer | logic |
| damage/injury | + | COLLAGEN | 29925522, 29340130 |
| RAC1 | + | Lamellipodia | 23121917, 16195235, 12453877 |
| ROCK1 | + | Stress fiber formation | 23121917 |
| Ca2+ | + | Stress fiber formation | 23121917 |
| Stress fiber formation | + | Shape Change | 23121917 |
| Lamellipodia | + | Shape Change | 23121917 |
| GP1b-IX-V | + | PI3K | 35615944 |
| GP1b-IX-V | + | PLC | 35615944 |
| vWF | + | GP1b-IX-V | 28228483, 35615944 |
| damage/injury | + | vWF | 28228483, 35615944 |

## Supplementary References

Balkenhol, J., Kaltdorf, K.V., Mammadova-Bach, E., Braun, A., Nieswandt, B., Dittrich, M., and Dandekar, T. (2020). Comparison of the central human and mouse platelet signaling cascade by systems biological analysis. BMC Genomics *21*, 897. 10.1186/s12864-020-07215-4.

Bastida, J.M., Benito, R., Lozano, M.L., Marin-Quilez, A., Janusz, K., Martin-Izquierdo, M., Hernandez-Sanchez, J., Palma-Barqueros, V., Hernandez-Rivas, J.M., Rivera, J., and Gonzalez-Porras, J.R. (2019). Molecular Diagnosis of Inherited Coagulation and Bleeding Disorders. Semin Thromb Hemost *45*, 695-707. 10.1055/s-0039-1687889.

Berndt, M.C., and Andrews, R.K. (2011). Bernard-Soulier syndrome. Haematologica *96*, 355-359. 10.3324/haematol.2010.039883.

Breitenbach T, Englert N, Osmanoglu O, Rukoyatkina N, Wangorsch G, Heinze K, et al. A modular systems biological modelling framework studies cyclic nucleotide signaling in platelets. J Theor Biol. 2022;550:111222.

Chen, Y., Zhong, H., Zhao, Y., Luo, X., and Gao, W. (2020). Role of platelet biomarkers in inflammatory response. Biomark Res *8*, 28. 10.1186/s40364-020-00207-2.

Corken, A., Russell, S., Dent, J., Post, S.R., and Ware, J. (2014). Platelet glycoprotein Ib-IX as a regulator of systemic inflammation. Arterioscler Thromb Vasc Biol *34*, 996-1001. 10.1161/ATVBAHA.113.303113.

Davi, G., and Patrono, C. (2007). Platelet activation and atherothrombosis. N Engl J Med *357*, 2482-2494. 10.1056/NEJMra071014.

Davizon-Castillo, P., McMahon, B., Aguila, S., Bark, D., Ashworth, K., Allawzi, A., Campbell, R.A., Montenont, E., Nemkov, T., D'Alessandro, A., et al. (2019). TNF-alpha-driven inflammation and mitochondrial dysfunction define the platelet hyperreactivity of aging. Blood *134*, 727-740. 10.1182/blood.2019000200.

Diseases, G.B.D., and Injuries, C. (2020). Global burden of 369 diseases and injuries in 204 countries and territories, 1990-2019: a systematic analysis for the Global Burden of Disease Study 2019. Lancet *396*, 1204-1222. 10.1016/S0140-6736(20)30925-9.

Estevez, B., and Du, X. (2017). New Concepts and Mechanisms of Platelet Activation Signaling. Physiology (Bethesda) *32*, 162-177. 10.1152/physiol.00020.2016.

Feil, R., Lehners, M., Stehle, D., and Feil, S. (2022). Visualising and understanding cGMP signals in the cardiovascular system. Br J Pharmacol *179*, 2394-2412. 10.1111/bph.15500.

Golebiewska, E.M., and Poole, A.W. (2013). Secrets of platelet exocytosis - what do we really know about platelet secretion mechanisms? Br J Haematol *165*, 204-216. 10.1111/bjh.12682.

Gu, S.X., and Dayal, S. (2020). Inflammation mediated platelet hyperactivity in aging. Ann Blood *5*. 10.21037/aob.2020.03.01.

Jamasbi, J., Megens, R.T., Bianchini, M., Munch, G., Ungerer, M., Faussner, A., Sherman, S., Walker, A., Goyal, P., Jung, S., et al. (2015). Differential Inhibition of Human Atherosclerotic Plaque-Induced Platelet Activation by Dimeric GPVI-Fc and Anti-GPVI Antibodies: Functional and Imaging Studies. J Am Coll Cardiol *65*, 2404-2415. 10.1016/j.jacc.2015.03.573.

Karl, S., and Dandekar, T. (2015a). Convergence behaviour and control in non-linear biological networks. Scientific reports *5*, 9746.

Karl, S., and Dandekar, T. (2015b). Convergence behaviour and Control in Non-Linear Biological Networks. Sci Rep *5*, 9746. 10.1038/srep09746.

Klavina, P.A., Leon, G., Curtis, A.M., and Preston, R.J.S. (2022). Dysregulated haemostasis in thrombo-inflammatory disease. Clin Sci (Lond) *136*, 1809-1829. 10.1042/CS20220208.

Li, Z., Delaney, M.K., O'Brien, K.A., and Du, X. (2010). Signaling during platelet adhesion and activation. Arterioscler Thromb Vasc Biol *30*, 2341-2349. 10.1161/ATVBAHA.110.207522.

Linkous, A., and Yazlovitskaya, E. (2010). Cytosolic phospholipase A2 as a mediator of disease pathogenesis. Cell Microbiol *12*, 1369-1377. 10.1111/j.1462-5822.2010.01505.x.

Lopez-Vilchez, I., Diaz-Ricart, M., White, J.G., Escolar, G., and Galan, A.M. (2009). Serotonin enhances platelet procoagulant properties and their activation induced during platelet tissue factor uptake. Cardiovasc Res *84*, 309-316. 10.1093/cvr/cvp205.

Lutsey, P.L., and Zakai, N.A. (2023). Epidemiology and prevention of venous thromboembolism. Nat Rev Cardiol *20*, 248-262. 10.1038/s41569-022-00787-6.

Mac Mullan, P.A., Peace, A.J., Madigan, A.M., Tedesco, A.F., Kenny, D., and McCarthy, G.M. (2010). Platelet hyper-reactivity in active inflammatory arthritis is unique to the adenosine diphosphate pathway: a novel finding and potential therapeutic target. Rheumatology (Oxford) *49*, 240-245. 10.1093/rheumatology/kep377.

Makhoul, S., Walter, E., Pagel, O., Walter, U., Sickmann, A., Gambaryan, S., Smolenski, A., Zahedi, R.P., and Jurk, K. (2018). Effects of the NO/soluble guanylate cyclase/cGMP system on the functions of human platelets. Nitric Oxide *76*, 71-80. 10.1016/j.niox.2018.03.008.

Mischnik, M., Gambaryan, S., Subramanian, H., Geiger, J., Schutz, C., Timmer, J., and Dandekar, T. (2014). A comparative analysis of the bistability switch for platelet aggregation by logic ODE based dynamical modeling. Mol Biosyst *10*, 2082-2089. 10.1039/c4mb00170b.

Muehlenkamp, J.J., and Brausch, A.M. (2019). Protective factors do not moderate risk for past-year suicide attempts conferred by recent NSSI. J Affect Disord *245*, 321-324. 10.1016/j.jad.2018.11.013.

Nagy, Z., and Smolenski, A. (2018). Cyclic nucleotide-dependent inhibitory signaling interweaves with activating pathways to determine platelet responses. Res Pract Thromb Haemost *2*, 558-571. 10.1002/rth2.12122.

Oury, C., Lecut, C., Hego, A., Wera, O., and Delierneux, C. (2015). Purinergic control of inflammation and thrombosis: Role of P2X1 receptors. Comput Struct Biotechnol J *13*, 106-110. 10.1016/j.csbj.2014.11.008.

Oved, J.H., Lambert, M.P., Kowalska, M.A., Poncz, M., and Karczewski, K.J. (2021). Population based frequency of naturally occurring loss-of-function variants in genes associated with platelet disorders. J Thromb Haemost *19*, 248-254. 10.1111/jth.15113.

Palma-Barqueros, V., Revilla, N., Sanchez, A., Zamora Canovas, A., Rodriguez-Alen, A., Marin-Quilez, A., Gonzalez-Porras, J.R., Vicente, V., Lozano, M.L., Bastida, J.M., and Rivera, J. (2021). Inherited Platelet Disorders: An Updated Overview. Int J Mol Sci *22*. 10.3390/ijms22094521.

Price, J., Lord, J.M., and Harrison, P. (2020). Inflammaging and platelet hyperreactivity: A new therapeutic target? J Thromb Haemost *18*, 3-5. 10.1111/jth.14670.

Raskob, G.E., Angchaisuksiri, P., Blanco, A.N., Buller, H., Gallus, A., Hunt, B.J., Hylek, E.M., Kakkar, A., Konstantinides, S.V., McCumber, M., et al. (2014). Thrombosis: a major contributor to global disease burden. Arterioscler Thromb Vasc Biol *34*, 2363-2371. 10.1161/ATVBAHA.114.304488.

Sharma, S., Tyagi, T., and Antoniak, S. (2022). Platelet in thrombo-inflammation: Unraveling new therapeutic targets. Front Immunol *13*, 1039843. 10.3389/fimmu.2022.1039843.

Stalker, T.J., Newman, D.K., Ma, P., Wannemacher, K.M., and Brass, L.F. (2012). Platelet signaling. Handb Exp Pharmacol, 59-85. 10.1007/978-3-642-29423-5_3.

Stark, K., and Massberg, S. (2021). Interplay between inflammation and thrombosis in cardiovascular pathology. Nat Rev Cardiol *18*, 666-682. 10.1038/s41569-021-00552-1.

Wallace, D.F. (2009). Die Entdeckung des Unendlichen: Georg Cantor und die Welt der Mathematik (Piper).

Wendelboe, A.M., and Raskob, G.E. (2016). Global Burden of Thrombosis: Epidemiologic Aspects. Circ Res *118*, 1340-1347. 10.1161/CIRCRESAHA.115.306841.

Yeung, J., Li, W., and Holinstat, M. (2018). Platelet Signaling and Disease: Targeted Therapy for Thrombosis and Other Related Diseases. Pharmacol Rev *70*, 526-548. 10.1124/pr.117.014530.
